# Conditional guide RNA through two intermediate hairpins for programmable CRISPR/Cas9 function: building regulatory connections between endogenous RNA expressions

**DOI:** 10.1093/nar/gkaa842

**Published:** 2020-10-17

**Authors:** Jiao Lin, Yan Liu, Peidong Lai, Huixia Ye, Liang Xu

**Affiliations:** MOE Key Laboratory of Bioinorganic and Synthetic Chemistry, School of Chemistry, Sun Yat-Sen University, Guangzhou 510275, China; MOE Key Laboratory of Bioinorganic and Synthetic Chemistry, School of Chemistry, Sun Yat-Sen University, Guangzhou 510275, China; MOE Key Laboratory of Bioinorganic and Synthetic Chemistry, School of Chemistry, Sun Yat-Sen University, Guangzhou 510275, China; MOE Key Laboratory of Bioinorganic and Synthetic Chemistry, School of Chemistry, Sun Yat-Sen University, Guangzhou 510275, China; MOE Key Laboratory of Bioinorganic and Synthetic Chemistry, School of Chemistry, Sun Yat-Sen University, Guangzhou 510275, China

## Abstract

A variety of nanodevices developed for nucleic acid computation provide great opportunities to construct versatile synthetic circuits for manipulation of gene expressions. In our study, by employing a two-hairpin mediated nucleic acid strand displacement as a processing joint for conditional guide RNA, we aim to build artificial connections between naturally occurring RNA expressions through programmable CRISPR/Cas9 function. This two-hairpin joint possesses a sequence-switching machinery, in which a random trigger strand can be processed to release an unconstrained sequence-independent strand and consequently activate the self-inhibitory guide RNA for conditional gene regulation. This intermediate processor was characterized by the fluorescence reporter system and applied for regulation of the CRISPR/Cas9 binding activity. Using plasmids to generate this sequence-switching machinery *in situ*, we achieved the autonomous genetic regulation of endogenous RNA expressions controlled by other unrelated endogenous RNAs in both *E. coli* and human cells. Unlike previously reported strand-displacement genetic circuits, this advanced nucleic acid nanomachine provides a novel approach that can establish regulatory connections between naturally occurring endogenous RNAs. In addition to CRISPR systems, we anticipate this two-hairpin machine can serve as a general processing joint for wide applications in the development of other RNA-based genetic circuits.

## INTRODUCTION

Nucleic acid computation is one of the dynamic nucleic acid nanotechnologies that can carry out analogue or digital computation to achieve information processing (1–5). Toehold-mediated strand displacement is the key driving force for the enzyme-free kinetics, which enables programmable and reversible switching of nucleic acid structures between different functional states (6–8). A variety of nucleic acid computers and circuits have been constructed in test tubes based on toehold-mediated strand displacement to perform kinds of tasks, such as acting as logic gates, making decisions, and transducing and amplifying signals (9–13). However, how to apply these powerful molecular computers to control biological functions or even create artificially biological regulations in living cells is continuously intriguing but challenging. These functional nanodevices can greatly promote the development of synthetic biology (3,4).

Constructing genetic circuits that can conditionally control gene expression to program cellular functions is a long-term goal in manipulation of biosystems (14). In contrast to other biological molecules, nucleic acid-based nanotechnology, taking advantage of highly specific and programmable Watson–Crick base pairing, is the most promising strategy to engineer versatile synthetic circuits in living organisms. Through the toehold-mediated strand displacement reaction, a number of synthetic RNA regulatory elements that can control transcriptional and translational response to cognate RNAs have been developed based on the riboswitch and RNA interference machinery (15–20). Moreover, by rational design and clustering multiple strand displacement events in mRNA riboregulators, the multiplex control of gene translation with complex logic function has been constructed in *Escherichia coli* (21–23). However, these riboregulators generally require additional engineering of target RNAs, making them difficult to directly manipulate the naturally occurring RNAs. Recently, the great development of CRISPR technology offers a new approach to control and program living organisms (24–27). The nuclease-dead Ca9 (dCas9) has been repurposed as effective regulatory tools that can inhibit (CRISPRi) or activate (CRISPRa) gene transcription by conjugating with proper effectors (28,29). This powerful technology can theoretically manipulate any gene expressions, whereas conditional control of CRISPR function is particularly essential for transformative applications. Plenty of attention has been attracted to develop all kinds of conditional control based on either engineering of Cas9 protein (30–37) or modifications of guide RNA (gRNA) (38–45). Recently, through the direct strand displacement of rationally designed gRNA, several studies have shed some light that target gene expressions could be programmed by trigger RNAs to conditionally activate CRISPR function (46–48). These investigations suggest the great potential for development of genetic circuits based on the combination of strand displacement with CRISPR technology.

**Figure 1.**
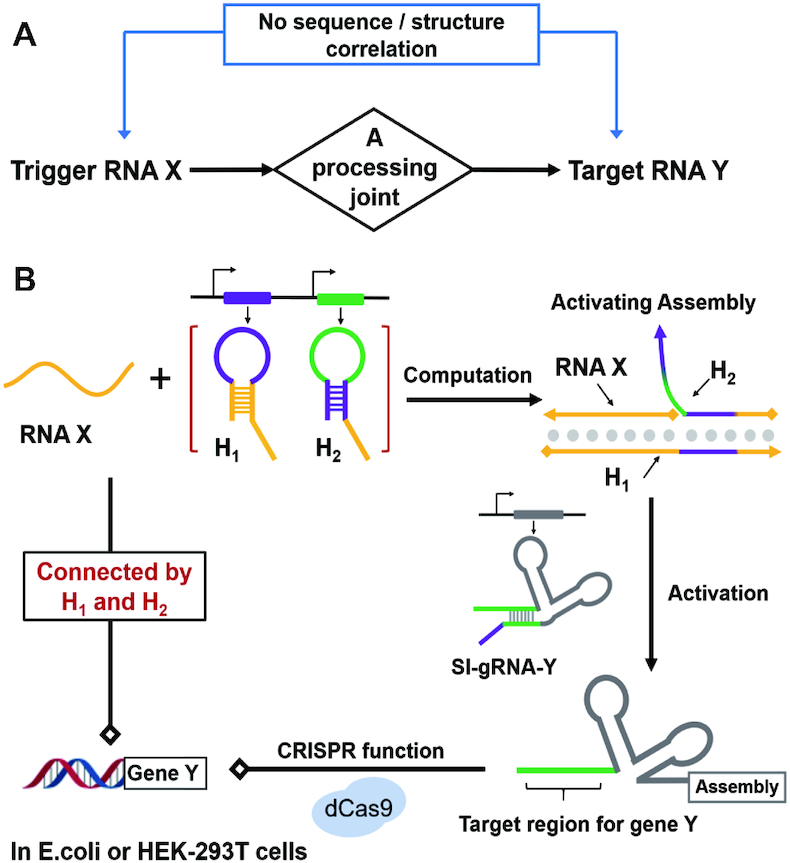
Conditional guide RNA with a two-hairpin mediated strand displacement as an RNA processing joint. (**A**) The concept for introducing a computing element as a joint between trigger and target RNA. (**B**) Schematics of the two-hairpin mediated nucleic acid computation in programming CRISPR/Cas9 function. The sequence-related strands are depicted by the same colour, and different colours indicate independent sequences of RNA strands. RNA X is sequence-independent of gRNA-Y and gene Y. After the two-hairpin processing, RNA X can activate the self-inhibitory gRNA-Y (SI-gRNA-Y) to regulate the gene Y.

Despite tremendous efforts to generate RNA circuits in living cells, the key structural switch of these circuits generally relies on the direct or combinatorial strand displacement (49) between the trigger and the target RNA, suggesting that the trigger strand must at least share partial sequence-dependence with the target RNA. Direct connection between the trigger and the target RNA can benefit their tight interaction, but the sequence design will inevitably encounter the lack of flexibility and independence. One can envision that if an intermediate RNA processing layer is introduced as a ‘joint’ between the trigger and target RNA, these two RNAs would not be necessary to directly contact with each other (Figure 1A). Consequently, both the trigger and the target can be fully independent and have no relationship in either the sequence or the structure. This independence is critically important if the RNA circuit is meant to establish connections between two unrelated endogenous genetic events. Therefore, the key element for separating the trigger and the target RNA is how to construct an intermediate joint as a processor that can link two fully independent RNA strands together. This RNA processor needs to convert the trigger strand into another desired strand, so that it can activate the function of target RNA that has no sequence-similarity with the initial one.

Herein, inspired by a variety of nanodevices developed for nucleic acid computation (1–5), we aimed to build an intermediate RNA processor in a CRISPR/Cas9-based gRNA circuit to link sequence-independent RNA expression events. Unlike direct strand displacement, we introduced an advanced nucleic acid machine as an RNA processing joint. This processor functioned through the two-hairpin mediated self-assembly that could release a dangled single strand without any sequence-dependence on the trigger strand. Therefore, the unrelated RNA X could be processed to activate the self-inhibitory gRNA-Y (SI-gRNA-Y) and consequently target RNA Y expression via CRISPR function (Figure 1B). By introducing the two-hairpin mediated nucleic acid computation in conditional gRNA, we successfully achieved autonomous control of endogenous gene expressions regulated by other independent endogenous RNAs through CRISPR/Cas9 function in both *E. coli* and human cells.

## MATERIALS AND METHODS

### Materials

All primers and DNA sequences used in this study including fluorophore-labeled strands were ordered from Sangon Biotechnology (Shanghai, China). Plasmid mini-prep kit, DNA gel extraction kit, PCR product purification kit, bacterial total RNA extraction kit, DEPC-treated water, isopropanol, ethanol, kanamycin sulfate, sodium chloride, yeast extract, tryptone, ampicillin, isopropyl β-d-thiogalactoside (IPTG), and agarose were purchased from Sangon Biotechnology. *E. coli* strain BL21(DE3) and DH5α (TransGen Biotech), Phanta Max Super-Fidelity DNA Polymerase, Hiscript III RT SuperMix for qPCR, T7 High Yield Transcription Kit, and miRNA first Strand cDNA Synthesis Kit were purchased from Vazyme (Nanjing, China). iTaq™ Universal SYBR Green Supermix was purchased from Bio-Rad. HEK-293T cells were obtained from Laboratory Animal Center of Sun Yat-sen University. Other experimental materials used in this study included Dulbecco's modified Eagle's medium (DMEM; Gibco), Penicillin–streptomycin (PS; Gibco), Fetal bovine serum (FBS; Gibco), Opti-medium (Gibco), Lipofectamine 3000 (Invitrogen), trypsin-EDTA (Life technology), Trizol (Life technology), phosphate buffer solution (PBS, Jinuo, Hangzhou, China), and Ni-NTA agarose (Thermo).

dCas9 protein was prepared and purified as described previously (50). Briefly, the coding sequence for dCas9 (nuclease-dead Cas9) was cloned into BamHI and HindIII site in a pET28a expression vector to construct pET-28a-dCas9 with an N-terminal hexa-histidine (6× His) tag. BL21(DE3) transformed with pET-28a-dCas9 was grown in LB medium supplemented with 50 μg/ml kanamycin at 37°C to reach OD 0.6. Then, the temperature was cooled down to 16°C, and the protein expression was induced with IPTG (0.25 mM) overnight (∼16 h). The protein was purified by Ni-NTA after lysis of cells in 20 mM Tris pH 7.4, 500 mM NaCl (supplemented with 10 mM β-mercaptoethanol, and 1 mM phenylmethylsulfonyl fluoride (PMSF, aladdin)). The purified protein was concentrated to ∼20 μM in 20 mM Tris–HCl (pH 7.4) with 500 mM NaCl, 1 mM DTT and 5% glycerol, flash-frozen in liquid nitrogen, and stored at −80°C.

The gRNA used in dCas9 binding assays were prepared by T7 *in vitro* RNA transcription. Briefly, the duplex DNA containing the T7 promoter and coding sequence of gRNA was prepared and purified by commercial PCR kits. The *in vitro* transcription reaction was performed by T7 High Yield Transcription Kit and incubated for 12 h at 37°C. After reaction, the DNA template was digested by DNase I, and the reaction mixture was subsequently processed by phenol extraction, followed by ethanol precipitation. The RNA precipitate was dissolved in DEPC-treated water and desalted through Micro Bio-Spin 6 Column (Bio-Rad). The eluate containing the gRNA transcript was stored at −20°C or ready for use immediately. The purity of *in vitro* transcribed RNA product was confirmed by denaturing PAGE.

### Plasmid construction

Important plasmids used in this study were listed in Supplementary Table S1. The plasmid pJ-dCas9 was constructed by cloning the promoter J23119, RBS and dCas9 into pET28a using EcoRI and XhoI sites. For the pJ-gRNA-dCas9 construction, a separate promoter J23119 and gRNA were cloned into pJ-dCas9. For the plasmid pJ-SH construction, SI-gRNA, Hairpin 1 and Hairpin 2 were cloned into the ampicillin-resistant plasmid under the control of three separate J23119 promotors. For the plasmid pJ-tri construction, trigger sequence under the control of J23119 promoter was cloned into the chloramphenicol-resistant plasmid using BamHI and XhoI sites. pJ-lac-Tri was constructed by cloning a 2× lac operator into plasmid pJ-tri using EcoRI and HindIII sites between the J23119 promoter and the trigger sequence. For the plasmid pU6-SH construction, SI-gRNA, Hairpin 1 and Hairpin 2 were cloned into the kanamycin-resistant plasmid under the control of three separate U6 promotors.

### Fluorescence measurement

The two hairpin strands (H_1_ and H_2_) and the reporter duplex RepF:RepQ (1: 1.5 ratio) were annealed separately in the stock buffer (20 mM Tris, pH 7.5, 140 mM NaCl). Hybridization occurred upon mixing of 60 nM trigger strand (T), 60 nM H_1_, 60 nM H_2_, and 30 nM reporter duplex RepF:RepQ in the reaction buffer (20 mM Tris–HCl pH 7.5, 140 mM NaCl, 5 mM MgCl_2_). The fluorescence was then measured after 20-min incubation at 37°C on a SHIMADZU-RF-6000 fluorescence spectrophotometer with the excitation wavelength at 480 nm and the emission range from 505 to 600 nm. For concentration-dependent fluorescence measurement, different concentrations of T strand were incubated with 60 nM H_1_ and 60 nM H_2_ in the presence of 30 nM RepF:RepQ duplex. The fluorescence signal was recorded as described above. For the kinetic measurements, 60 nM H_1_, 60 nM H_2_ and 30 nM RepF:RepQ duplex were pre-mixed in the reaction buffer. The hybridization started upon the addition of different concentrations of T strand, and the fluorescence signal was then monitored in a kinetic mode with the excitation wavelength at 480 nm and the emission at 520 nm. All kinetic measurements were carried out at 37°C. Sequences used in this experiment was listed in Supplementary Table S2.

### DNA binding assay by dCas9

The duplex DNA substrate was labelled by 5′-FAM to monitor the gel shift behaviour in the presence of different gRNAs and computing conditions to check the binding ability of dCas9. To investigate the effect of 5′-SI-gRNA and 3′-SI-gRNA on binding ability of dCas9, the in vitro transcribed 5′-10/12/15-SI-gRNA and 3′-10/12/15-SI-gRNA were incubated with FAM-dsDNA (100 nM) and dCas9 protein (1 μM) in the binding buffer (20 mM Tris–HCl pH 7.5, 100 mM KCl, 1 mM DTT, and 0.1 mM EDTA) at 37°C for 10 min. The dCas9 binding products were then analysed by electrophoresis on 5% polyacrylamide gel on ice, and imaged by Gel Image System (Tanon 2500R).

To illustrate how the two-hairpin computing process regulated the dCas9 binding ability *in vitro*, firstly, self-inhibitory RNA (SI-gRNA, 1 μM) was incubated in the presence or absence of the trigger strand (2 μM) or computing hairpins (H_1_+H_2_, 2 μM) at 37°C for 1 h in the binding buffer. The pre-incubated SI-gRNA/trigger/Hairpins were then mixed with FAM-dsDNA (100 nM) and dCas9 protein (1 μM), and further incubated at 37°C for 10 min. The dCas9 binding products were analysed as described above. To study concentration-dependent activation of SI-gRNA, different concentrations of trigger strand (0, 0.25, 0.5, 1 and 2 μM, respectively) were incubated with the mixture of computing hairpins (H_1_+H_2_, 2 μM) and SI-gRNA (1 μM) in the binding buffer at 37°C for 1 h, followed by addition of FAM-dsDNA (100 nM) and dCas9 protein (1 μM) with another 10 min incubation at 37°C. The dCas9 binding products were analysed as described above. For the annealing experiment, 1 μM 3′-SI-gRNA was annealed with the excessive trigger strand and hairpins (2 μM) to form the defined assembly, followed by a 10-min incubation with FAM-dsDNA (100 nM) and dCas9 protein (1 μM) at 37°C. The dCas9 binding products were analysed as described above.

To verify the influence of 3′-extension or 5′-extension of gRNA on binding capacity of dCas9, modified gRNAs with either 3′-overhang or 5′-overhang were firstly paired with a long duplex structure (59 bp) at 37°C for 1 h in the binding buffer. The pre-incubated modified gRNAs with long tails were then mixed with FAM-dsDNA (100 nM) and dCas9 protein (1 μM), and further incubated at 37°C for 10 min. The dCas9 binding products were analysed as described above. All DNA and RNA sequences used in the binding assay are listed in Supplementary Table S3.

### Transcriptional regulation in *E. Coli*

To investigate transcriptional repression controlled by exogenous trigger strands, DH5α cells were co-transformed with plasmids pJ-SH (expressing SI-gRNA and correct/incorrect computing hairpins), pJ-dCas9 (expressing dCas9 for CRISPRi), and pJ-trigger (expressing trigger strand) by heat shock. Single colonies were picked from agar plates and grown in LB medium (10 g/l tryptone, 5 g/l yeast extract,10 g/l NaCl) supplemented with 100 μg/ml ampicillin, 25 μg/ml chloramphenicol and 50 μg/ml kanamycin for ∼10–12 h at 37°C. The cultured bacterial cells were then collected to obtain total RNA. For the IPTG-induced expression of trigger strand, pJ-lac-trigger (expressing trigger strand induced by IPTG) was co-transformed to replace pJ-trigger. The transformed cells were cultured under the same conditions to reach OD ∼1 as described above. Expression of the exogenous trigger strands were induced by inoculating fresh LB media containing the 500 mM IPTG at an initial OD ∼0.03. When reaching OD ∼1, the samples were collected to obtain total bacterial RNA. Related sequence information was listed in Supplementary Table S4.

To achieve sRNA induced endogenous gene repression (see Supplementary Table S5 for selected sRNA sequences), DH5α were co-transformed with plasmids pJ-SH (expressing SI-gRNA_g_, SI-gRNA_L_ or SI-gRNA_R_, and correct or incorrect hairpins) and pJ-dCas9 (expressing dCas9 for CRISPRi). Single colonies were picked from agar plates and grown in LB medium supplemented with 100 μg/ml ampicillin and 50 μg/ml kanamycin. The cultured bacterial cells were then collected to obtain total RNA. For the experiment of 2,2′-bipyridyl induced repression of galA gene, the induction of RyhB sRNA was controlled by inoculating fresh LB media containing 0.6 mM of 2,2′-bipyridyl at an initial OD ∼0.03. When reached OD ∼1, the samples were collected to obtain total bacterial RNA. As control, the sample without treatment of 2,2′-bipyridyl was processed by the same procedure. Related sequence information was listed in Supplementary Table S6.

### qPCR for determination of RNA expression levels in *E. coil*

To obtain bacterial RNA, cell samples were pelleted by centrifugation at 3000 g for 5 min, and washed twice with PBS. Then total RNA was extracted from samples immediately using bacterial total RNA Rapid extraction kits (Sangon Biotechnology) according to manufacturer's instruction. Extracted total RNA was quantified by the UV–Vis spectrophotometry and the value of OD_260/280_ was between 1.8 and 2.0.

For cDNA synthesis, the total RNA was reverse transcribed using cDNA Synthesis Kit (Vazyme). Briefly, 1 μg total RNA was mixed with the RT SuperMix (containing dNTP, reaction buffer, reverse transcriptase, RNase inhibitor and random primers) in a total reaction volume of 20 μl. Reverse transcription (RT) was conducted at 37°C for 15 min to obtain cDNA, followed by the inactivation of the reverse transcriptase at 85°C for 15 s. All RT reactions were performed on the T100 Thermal cycler (Bio-Rad).

The qPCR experiments were carried out in the reaction mixture containing SYBR Green Supermix (Bio-Rad), 0.2 μM forward primer, 0.2 μM reverse primer and diluted cDNA using the CFX96 Real-Time system (Bio-Rad). Reference gene (rssA, 16S RNA) was used to normalize the lacZ, galA and MicF expression levels. The thermocycling condition for the lacZ and galA gene was: 95°C for 10 min followed by 40 cycles of 95°C for 15 s and 62°C for 1 min. The thermocycling condition for MicF and rssA was: 95°C for 10 min followed by 40 cycles of 95°C for 15 s and 58°C for 1 min. The purity of the PCR product was validated by the electrophoresis gel and the melting profile. The qPCR Data were then analysed using the Bio-Rad CFX maestro Real-Time PCR analysis software. The cycle-threshold (Ct) values were determined by default settings of the software. The qPCR results were only determined as effective data when the Ct values were <35. Reference gene was used to normalize expression levels using the 2^−ΔΔCT^ method. Three technical replicates were performed for each PCR reaction. The sequence information of the primers used in qPCR was listed in Supplementary Table S7.

### Lentiviral packaged HEK-293T cells

DNA sequence encoding dCas9 (D10A and H840A) with VP64-p65-Rta (VPR) and EGFP fused to its C-terminus was cloned into pHR-SFFV (Addgene #79121) lentiviral vector to generate pHR-SFFV-dCas9-VPR-EGFP for constitutive expression of dCas9-VPR-EGFP. To obtain Viral production cells, 1 000 000 cells were seeded into six-well plates before co-transfection of 1000 ng pMD2.G (Addgene #12259), 2000 ng pCMV-dR8.2 (Addgene #84550) and 3000 ng pHR-SFFV-dCas9-VPR-EGFP using 16 μl P3000 and 7 μl Lipofectamine 3000. The transfection medium was then changed to 2 ml fresh medium after 6-h incubation. Twenty-four hours later, the culture medium was collected and filtered to remove cells, and treated with 10 μg/μl 1.6 μl polybrene before infecting the freshly seeded 293T cells with a density of 20 000 cells in a six-well plate. The infected cells were then collected and sorted by flow cytometer (Beckman moFlo XDP) to obtain 293T-dCas9-VPR cells.

### Transcriptional regulation in HEK-293T cells

To achieve miRNA (see Supplementary Table S8 for selected miRNA sequences) induced transcriptional regulation of the CXCR4 and ASCL1 gene, 250 000 293T-dCas9-VPR cells were seeded in six-well plates, and immediately transfected with 2000 ng of p-U6-SH encoding SI-gRNA and the corresponding computing hairpins using 5.5 μl Lipofectamine 3000 and 4 μl P3000. For control experiment, 2000 ng p-U6-gRNA encoding the standard gRNA to target the CXCR4 or ASCL1 gene or without any target gene was also transfected using 5.5 μl Lipofectamine 3000 and 4 μl P3000. Cells were then collected for determination of RNA expression levels after a 2-day culture. Related sequence information was listed in Supplementary Tables S9 and S10.

Total RNA from 293T cells was isolated using trizol, chloroform, isopropanol and ethyl alcohol, and quantified by the UV–Vis spectrophotometry and the value of OD_260/280_ was between 1.8 and 2.0. For the reverse transcription of mRNA, 1 μg total RNA was used to generate cDNA through the poly(dT) primer using cDNA Synthesis Kit (Vazyme) according to manufacturer's instructions as described above.

The qPCR experiments were carried out in the reaction mixture containing SYBR Green Supermix (Bio-Rad), 0.2 μM forward primer, 0.2 μM reverse primer and diluted cDNA using the CFX96 Real-Time system (Bio-Rad). The thermocycling condition was: 95°C for 10 min followed by 40 cycles of 95°C for 15 s and 62°C for 1 min. The CXCR4 and ASCL1 mRNA levels were normalized using the expression level of GAPDH as control. The purity of the PCR product was validated by the electrophoresis gel and the melting profile. The qPCR results were analysed as described above. The sequence information of the primers was listed in Supplementary Table S11.

### miRNA reverse transcription and qPCR quantification

For cDNA synthesis of miRNA, reverse transcription was carried out using the miRNA reverse transcription kit (Vazyme) with the miR17/miR16/let-7a/U6 stem-loop primer (Supplementary Table S12). The primer was designed using miRNA Design V1.01 software. Briefly, 1 μg total RNA collected from 293T cells was mixed with 100 nM stem–loop primer and the RT SuperMix (containing dNTP, reaction buffer, reverse transcriptase and RNase inhibitor). The reaction was conducted at 25°C for 5 min and the 50°C for 15 min, followed by the inactivation of the reverse transcriptase at 85°C for 5 min. The qPCR experiments were carried out in the reaction mixture containing SYBR Green Supermix (Bio-Rad), 0.2 μM forward primer, 0.2 μM reverse primer and diluted cDNA using the CFX96 Real-Time system (Bio-Rad). The thermocycling condition was: 95°C for 10 min followed by 40 cycles of 95°C for 15 s and 60°C for 1 min. The U6 small nuclear RNA was used as the reference gene to normalize the miR17/miR16/let-7a expression level. The purity of the PCR product was validated by the electrophoresis gel and the melting profile. The qPCR results were analysed as described above. The sequence information of the primers was listed in Supplementary Table S13.

## RESULTS

### Design of nucleic acid intermediate joint to achieve sequence switch

**Figure 2.**
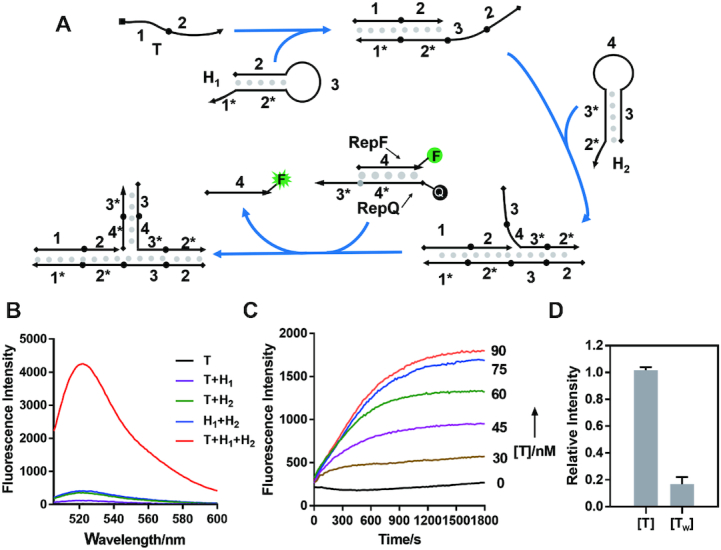
Characterization of the two-hairpin intermediate joint by fluorescence. (**A**) Workflow of the two-hairpin intermediate joint in the fluorescence reporter system. (**B**) Fluorescence measurement in the presence of different components after a 20-min incubation. The concentrations for these components: 60 nM trigger strand (*T*), 60 nM H_1_, 60 nM H_2_, and 30 nM reporter duplex RepF:RepQ. (**C**) Kinetic analysis of the computing process under different concentrations of trigger strand (0, 30, 45, 60, 75 and 90 nM, respectively). (**D**) Comparison of fluorescence signals between the correct (*T*) and incorrect (*T*_w_) trigger strand after a 24-h incubation. Error bars represent standard deviations derived from at least three independent experiments. Sequences used in this fluorescence assay were listed in Supplementary Table S2.

First, we need to design an effective nucleic acid intermediate joint that can achieve a fully independent sequence switch. In this design, the input sequence must share no sequence-dependence with the output sequence. Moreover, in order to achieve efficient and convenient sequence processing in living cells, this nucleic acid intermediate joint must be capable of being genetically expressed and generated *in situ*. Given that the hairpin structures can be easily formed during genetic expression and have been widely utilized in RNA switch, we decided to design the sequence-switching machine based on hairpins. The general designing principle of the hairpin structure was described in Supplementary Figure S1. As shown in Figure 2A, in our intermediate joint, the trigger strand (sequence **1–2**) firstly hybridizes with Hairpin-1 (H_1_) through a toehold-mediated strand displacement to release a strand **2–3**, followed by the opening of Hairpin-2 (H_2_) to generate an unconstrained single strand **3–4**. Notably, the dangled strand **3–4** has no sequence similarity with the initial input strand **1–2**. As a result, the trigger strand can be successfully switched into a fully independent strand. This newly released single strand is then capable of activating the target that is supposed to be unrelated to the trigger strand. Herein, to monitor the sequence-switching process, we employed a fluorescence reporter system as the target. To confirm the principle of this design, we utilized DNA sequences as a model system due to the ease of sample preparation, but the strand displacement and switching has no essential difference in the RNA system. Detailed sequence information was described in Supplementary Information.

As shown in Figure 2B, a single trigger strand could not induce the fluorescence enhancement due to no sequence similarity between the trigger (sequence **1–2**) and the target (sequence **3–4**). In the absence of the computing hairpin H_1_ or H_2_, the sequence switch could not be successfully achieved either as evidenced by no significant fluorescence enhancement. The fluorescence signal was released only when both the trigger and two computing hairpins co-existed, revealing that the activating strand against the reporter system was successfully generated by the two-hairpin processing joint. Moreover, the concentration-dependent analysis (Supplementary Figure S2) suggested the fluorescence signal was gradually enhanced along with the increasing concentration of trigger strand, indicating a positive concentration dependence upon the trigger strand during the sequence switch. We also performed the kinetic experiments in 37°C to monitor the fluorescence enhancement (Figure 2C), and these results showed that the fluorescence signal was saturated in tens of minutes under different concentrations of the trigger strand, demonstrating a prompt responding process. In addition, considering random single strands would co-exist with the two hairpins for a long time under the cellular conditions, we further verified the specificity of the two-hairpin intermediate joint with a 24-h incubation in the presence of a mismatched trigger strand. As shown in Figure 2D, the result exhibited a great discrimination between the correct and incorrect trigger strand. Taken together, our fluorescence reporter system indicated the two-hairpin intermediate joint can function as a sequence switch to link two independent strands.

### Activation of SI-gRNA by the two-hairpin computing assembly *in vitro*

Using this two-hairpin design, we next examined whether the binding activity of dCas9 can be effectively controlled by structural switch of the gRNA in a biochemical basis, given that the gene regulation relies on the specific binding of dCas9 protein. Here, we engineered the gRNA to make it initially stay in a self-inhibitory mode (SI-gRNA), in which the guide region was partially paired by a complementary sequence extended from either 5′- or 3′-end of the standard gRNA. To make sure a potent inhibition, the complementary sequence was designed to pair from the seed region of gRNA with a toehold left for strand displacement. As shown in Supplementary Figure S3, SI-gRNA with either a 5′- or 3′ extended complementary sequence (5′-SI-gRNA or 3′-SI-gRNA) against the guide sequence could cause strong inhibitory effect to prevent the binding of dCas9 with target DNA. Strand hybridization between the activating strand and the extended sequence initiated by the designed toehold would release the guide region for targeting. With the design of SI-gRNA, we then tested whether an independent strand that had no sequence similarity could activate the SI-gRNA through our two-hairpin intermediate joint. Similarly, we utilized DNA strands as the trigger and computing hairpins to activate SI-gRNA due to the ease of sample preparation, but the biochemical regulation of dCas9 binding activity would not be affected.

**Figure 3.**
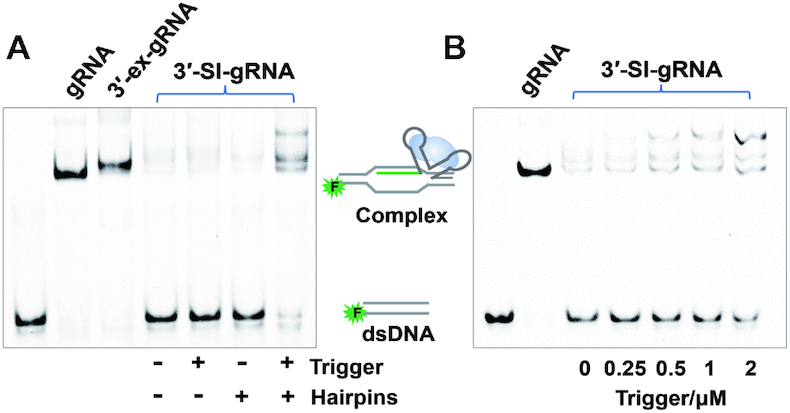
*In vitro* regulation of dCas9 binding activity mediated by the two-hairpin intermediate joint. (**A**) Gel shift for the formation of dCas9 binding complex controlled by an independent trigger strand to activate 3′-SI-gRNA. The complex formed with the standard gRNA was compared as control. The 3′-ex-gRNA represents the 3′ extended gRNA without complementary sequence against the guide region. The concentrations for these components: 100 nM dsDNA, 1 μM gRNA, 1 μM dCas9, 2 μM hairpins (H_1_&H_2_) and 2 μM trigger strand. (**B**) Concentration-dependent analysis of the formation of dCas9 binding complex. The concentration of trigger strand was 0, 0.25, 0.5, 1 and 2 μM, respectively. The dsDNA was labelled by the FAM fluorophore for visualization of the gels. Sequences used in this binding assay were listed in Supplementary Table S3.

Taking a 3′-SI-gRNA as a test in Figure 3A, we found that in the absence of computing hairpins, the trigger strand could not directly activate SI-gRNA due to its uncorrelated sequence design. Besides, without the trigger strand, the two computing hairpins alone could not release the gRNA activity either, as evidenced by no significant formation of the binding complex in the gel shift. The binding capacity of dCas9 could only be recovered in the presence of both the trigger and hairpins (Figure 3A), which was consistent with the fluorescence reporter system, suggesting the successful regulation of dCas9 binding activity by a sequence independent strand. We also monitored the concentration-dependent activation of 3′-SI-gRNA to control the binding behaviour of dCas9. As shown in Figure 3B, with the increased concentrations of the trigger strand, the formation of binding complex was gradually enhanced, revealing an effective regulation connected through the two-hairpin intermediate joint. Notably, instead of the single complex band in the gel shift, multiple complex bands were observed when the 3′-SI-gRNA was activated by the trigger and hairpins. To exclude the possibility that non-specific binding at the 3′-end of gRNA might induce the formation of multiple bands, the trigger strand, two hairpins and 3′-SI-gRNA were annealed together to form the defined assembly. As a result, multiple complex bands were still observed (Supplementary Figure S4). Herein, we speculated that these multiple complex bands would be likely caused by structural variation between the dCas9 protein and the activating assembly paired at the 3′-end of gRNA. Nevertheless, the complex formation of dCas9-RNA-DNA can be controlled by the sequence independent trigger strand.

Intriguingly, the SI-gRNA with 3′-extension (Figure 3) was more easily recovered than that with 5′-extension (Supplementary Figure S5) in terms of dCas9 binding behaviours. This could be attributed to the uneven tolerance of dCas9 to engineered gRNAs with the elongated 5′- or 3′-end (Supplementary Figure S6), which suggested that rather than the engineering of 5′-end, introducing extra regulatory nucleic acid elements in the 3′-end of gRNA is probably more suitable to minimize the negative impact on the dCas9 binding activity. Hence, we decided to utilize the 3′-extention to design the self-inhibitory gRNA (see Supplementary Figure S7 for the designing principle) and check its conditional response in the following cellular experiments.

### Employing an exogenous trigger strand to activate transcriptional repression in *E. coli*

**Figure 4.**
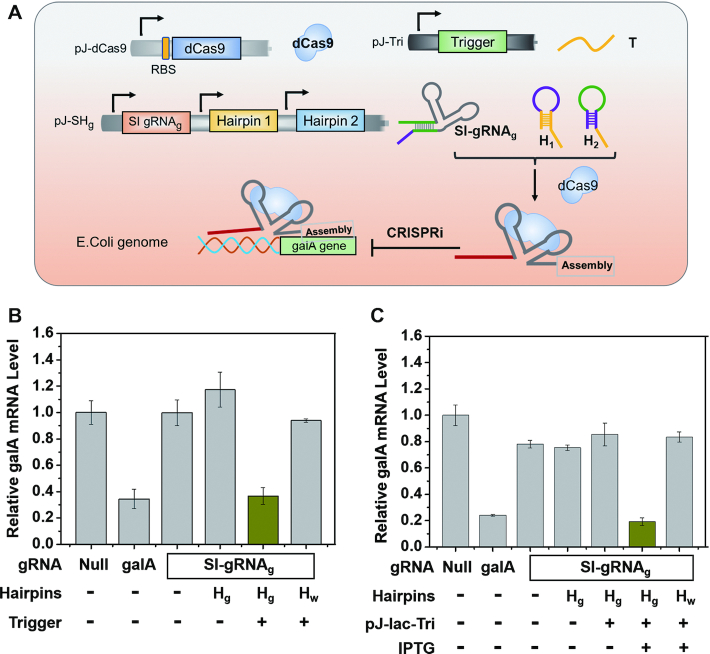
An independent exogenous RNA to control the expression of an endogenous gene in *E. coli*. (**A**) Designed schematics for the genetic circuits in *E. coli* to program CRISPRi function. (**B**) mRNA expression levels of galA gene controlled by the exogenous RNA. (**C**) mRNA expression levels of galA gene regulated by the IPTG-induced exogenous RNA expression. The expression levels of mRNA were determined by qPCR. gRNA that could not target any genes was selected as the negative control (Null); the standard gRNA that could directly target galA gene was selected as the positive control (galA). H_g_ represents the two computing hairpins to link the trigger RNA with galA gene, whereas H_w_ represents the wrong hairpins that could not activate the computing process. pJ-lac-Tri indicates the plasmid for the induced expression of trigger RNA controlled by IPTG. Error bars represent standard deviations derived from at least three biological replicates. Related sequence information in this figure was listed in Supplementary Table S4.

Next, we introduced the two-hairpin intermediate joint in *E. coli* to examine whether the CRISPRi could be activated by a sequence-independent RNA in the cellular context. All of exogenous RNAs including the computing hairpins, the artificial trigger strand and SI-gRNA were expressed by plasmids, allowing all the RNA computing procedures to occur *in situ*. We assembled the expression cassettes for self-inhibitory gRNA and two hairpin RNAs into a single plasmid, and placed dCas9 gene into another plasmid. Firstly, we verified whether SI-gRNA could be activated by an independent exogenous RNA, and to this end, we constructed a third plasmid that can express the trigger strand (Figure 4A). As shown in Figure 4B, the standard gRNA was designed to target the endogenous gene galA with strong inhibition on the transcriptional level in *E. coli*, whereas the SI-gRNA could not cause significant effect, even when the two computing hairpins were introduced into the expression plasmid, indicating the SI-gRNA can maintain a steadily inhibitory mode in absence of the designed trigger RNA. The SI-gRNA could only be activated when the trigger strand was expressed along with correct hairpins in *E. coli*, as illustrated by the released inhibitory effect on the galA transcription (Figure 4B). Importantly, our designed trigger RNA had no sequence similarity with either the gRNA or the target gene galA, but could regulate the expression of galA through through the two-hairpin intermediate joint. More than the galA gene, we could also use the same trigger RNA to control another endogenous lacZ gene expression by designing the corresponding two hairpins and SI-gRNA (Figure S8). These data indicated that through introducing a two-hairpin machine as a processing joint, an unrelated trigger RNA can activate the sequence-independent SI-gRNA to target endogenous genes with great flexibility in our design.

Furthermore, we utilized the *lac* operator for controlled expression of trigger RNA to regulate the endogenous galA RNA level (Figure 4C). The plasmid expressing the trigger RNA was under the control of the *lac* operator. In the absence of isopropyl β-d-thiogalactoside (IPTG), the trigger RNA failed to be expressed, and the two-hairpin intermediate joint could not be initiated, resulting in unaffected galA expression. Addition of IPTG induced the expression of trigger RNA, subsequently started the two-hairpin processing machine and eventually activated the CRISPRi function to target galA gene (Figure 4C). However, if the computing hairpins were not the correct pair, induction of trigger RNA would not cause any influence on the expression of galA gene. The significant control of galA expression upon the induced expression of the independent trigger RNA explicitly demonstrated the effectiveness and potency of the designed two-hairpin machine generated *in situ*.

### Endogenous sRNA induced endogenous gene repression in *E. coli*

**Figure 5.**
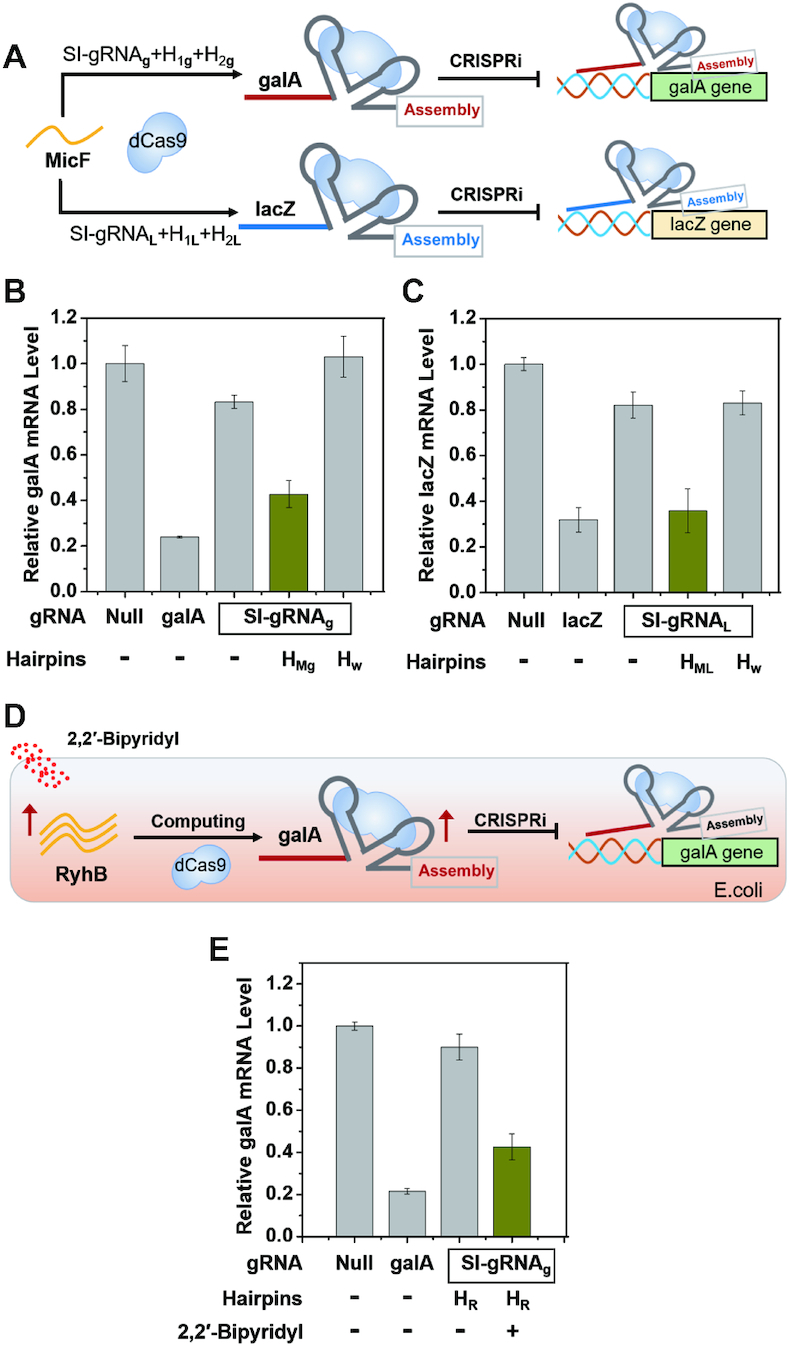
Automatous control of endogenous gene expression by independent endogenous RNA in *E. coli*. (**A**) The endogenous small RNA MicF could be processed by two different computing hairpins to control the expression of different endogenous genes (galA and lacZ). mRNA expression levels of galA (**B**) and lacZ (**C**) gene were regulated by MicF RNA. (**D**) Automatous control of the galA RNA expression by variation of an independent RyhB RNA level depicted by the genetic flow upon treatment of 2,2′-bipyridyl. (**E**) Expression level of the galA gene before and after treatment of 2,2′-Bipyridyl. The expression levels of mRNA were determined by qPCR. Standard gRNAs that could directly target galA or lacZ gene were selected as the positive control (galA/lacZ). H_Mg_ and H_ML_ represent computing hairpins to link MicF RNA with galA and lacZ gene, respectively. H_w_ represents the wrong hairpins that could not activate the computing process. H_R_ represents the computing hairpins to link RyhB RNA with galA gene. Error bars represent standard deviations derived from at least three biological replicates. Related sequence information in this figure was listed in Supplementary Table S6.

Since an exogenous trigger RNA expressed by a plasmid can regulate an independent endogenous gene, we further pursued whether the endogenous RNA could also be utilized to trigger CRISPR/Cas9 function to target another endogenous gene. We then designed a two-hairpin intermediate joint that could be activated by endogenous small RNA MicF in *E. coli* (Figure 5A). As output genes, we targeted either galA or lacZ gene with MicF RNA by the corresponding intermediate joint. Due to independence between the trigger and target RNAs in our system, the SI-gRNAs used for regulation of the galA and lacZ gene could maintain the same as the aforementioned, suggesting a highly flexible design with the computing hairpins as a processing joint. As shown in Figure 5B and C, the SI-gRNA could not be activated in the absence of computing hairpins or with the incorrect hairpins. Only in the presence of the correct two-hairpin joint, the MicF RNA could activate the corresponding SI-gRNA to repress either galA or lacZ gene expression (Figure 5B and C), and consequently the expression level of galA or lacZ RNA was directly linked to the originally unrelated MicF RNA, leading to the introduction of new genetic connections in *E. coli*.

A unique feature for connecting two endogenous RNAs is the output RNA expression could be controlled autonomously by variations of input RNA levels. In this scenario, the change of one endogenous RNA level would influence another independent endogenous RNA expression. Since the expression of MicF RNA could induce the repression of lacZ gene through the two-hairpin intermediate joint (Figure 5C), one would envision that if the MicF RNA level was reduced, the lacZ gene could be upregulated compared to the repressed state. Indeed, upon the MicF knockdown, significant upregulation of lacZ RNA was observed only in the presence of the correct two-hairpin joint (Supplementary Figure S9). To further confirm autonomous response of endogenous RNA, we also built an intermediate joint to connect the galA gene with a fully unrelated RyhB in *E. coli*. The expression of RyhB RNA was induced by iron deficiency (51), which could be controlled by addition of 2,2′-bipyridyl (Figure 5D) (21). Treatment of 2,2′-bipyridyl increased RyhB gene expression, subsequently triggered the two-hairpin machine, and consequently activated the SI-gRNA to regulate the galA gene. Eventually, the expression level of the output gene galA was greatly inhibited as shown in Figure 5E, which was originally supposed to be unrelated to iron deficiency (Supplementary Figure S10). Hence, by introducing the two-hairpin intermediate joint, we established an internal connection between originally independent endogenous genes RyhB and galA RNAs, in which the gene expression of galA was autonomously influenced by variations of RyhB RNA levels. These results together confirmed that introduction of the two-hairpin machine as a processing joint in the CRISPR/Cas9 function can establish an endogenous RNA link.

### Endogenous miRNA induced endogenous gene activation in HEK-293T cells

**Figure 6.**
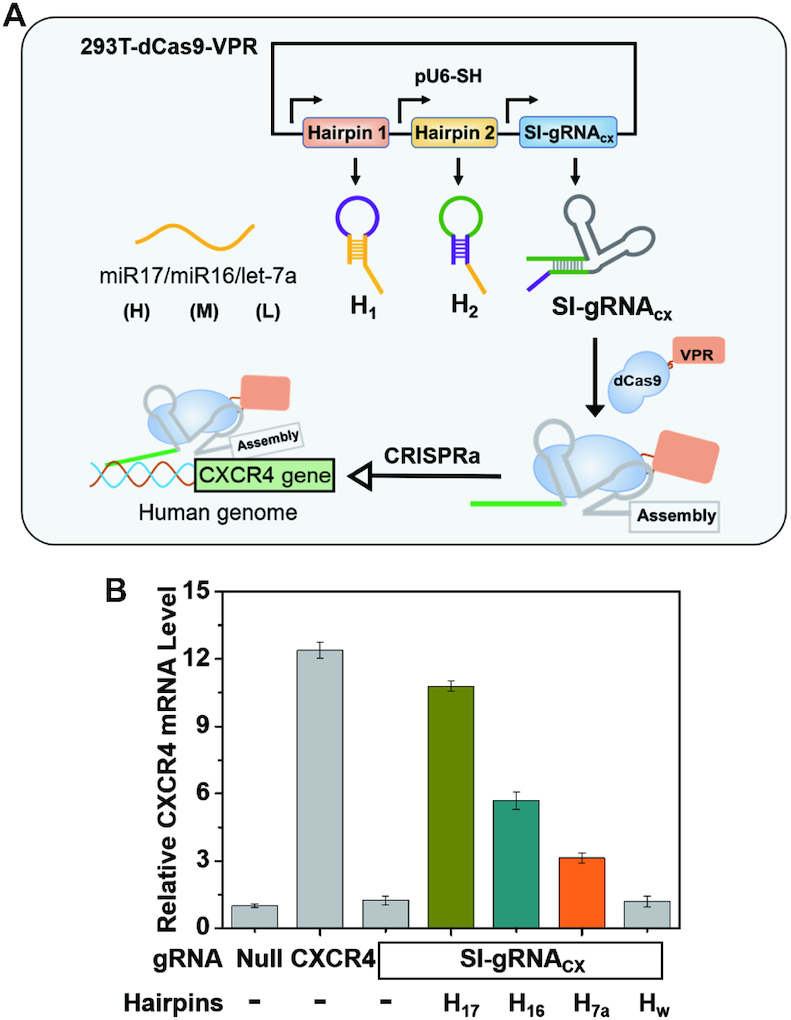
Establishing regulations between miRNAs and the unrelated CXCR4 gene in human cells. (**A**) Designed schematics for the genetic circuits in HEK-293T cells to link miRNA levels with the CXCR4 gene expression. ‘H’, ‘M’ and ‘L’ indicate the relative expression levels (high, medium, and low) of three selected miRNAs in HEK-293T cells, respectively. (**B**) Relative expression levels of the CXCR4 gene when linked with different expression levels of miRNAs. The expression levels of CXCR4 mRNA were determined by qPCR. The standard gRNA that could directly target CXCR4 gene was selected as the positive control (CXCR4). H_17_, H_16_ and H_7a_ represent computing hairpins for linking miR17, miR16 and let-7a miRNAs to the CXCR4 gene, respectively. H_w_ represents the wrong hairpins that could not activate the computing process. Error bars represent standard deviations derived from at least three biological replicates. Related sequence information in this figure was listed in Supplementary Table S9.

Having demonstrated our two-hairpin mediated CRISPR/Cas9 circuit can build endogenous RNA connections in *E. coli*, we further tested whether the same strategy could also function in mammalian cells. We prepared a lentivirus infected HEK-293T cell line with stably expressed dCas9-VP64-p65-Rta (dCas9-VPR) protein that could be used for CRISPR activation. A single plasmid including expression cassettes for both SI-gRNA and two hairpin RNAs was constructed for generating the intermediate joint in HEK-293T cells. In this experiment, we selected naturally occurring miRNA as the trigger strand to control another endogenous CXCR4 RNA expression as a proof of concept. We designed the corresponding computing elements to link three different miRNAs (miR17, miR16 and let-7a) with the CXCR4 gene (Figure 6A). Only when transfecting the plasmid with the correct computing hairpins, the SI-gRNA was activated via miRNAs and subsequently upregulate the expression level of CXCR4 through CRISPRa function (Figure 6B). Intriguingly, these miRNAs exhibited different levels in HEK-293T cells (Supplementary Figure S11), and as a result, the high expression level of miRNAs (miR17) could induce more enhancement of CXCR4 activation than the less abundant miRNA (let-7a), which confirmed that our designed two-hairpin intermediate joint can respond to the variations of RNA expression levels in mammalian cells as well. In addition to the CXCR4 gene, we also utilized the high abundant miR17 as a trigger to target another endogenous gene, ASCL1, through the two-hairpin machine (Supplementary Figure S12), and similar results were also observed. Notably, the transcriptional activation on the ASCL1 gene was relatively less effective than that on CXCR4, which might be attributed to different sensitivities towards the released CRISPR/Cas9 function in the different positions and environments of chromosomes.

## DISCUSSION

Kinds of sophisticated nanomachines based on nucleic acid computation have been greatly developed in the past few years, whereas how to apply these complexed computing systems in living cells is still challenging. A common feature between nucleic acid computation and gRNA in CRISPR is that both of them functioned through predictable Watson-Crick base pairing. Hence, conditional control of gRNA could be naturally coupled with nucleic acid computation. Recent applications of nucleic acid computation for developments of conditional gRNA for gene regulation (46–48), as well as other RNA-based circuits, such as riboregulators (21–23) and siRNA systems (15–17), were generally based on the simple toehold-mediate strand displacement between the trigger and the target RNA. This would face an inevitable issue that the trigger RNA (either single strand or the combinatory strand) must share at least partial sequence-dependence with the target RNA to achieve the strand displacement and subsequent activation, making the trigger RNA highly dependent on the target RNA. In some reported studies, engineering of gRNA had to move the dependent sequence into the futile region to avoid the correlation with the functional region for desired purposes (47,52,53), but it would leave great limitations for the flexibility of design. To overcome this sequence constraint, we employed an advanced computing procedure that one strand can be processed through a two-hairpin system to release another sequence-independent strand. We utilized this two-hairpin intermediate joint to control the activation of the self-inhibitory gRNA for targeting the designed gene by a fully unrelated trigger RNA. Since the trigger and the target RNA were uncorrelated in our system, selection and designing of these RNAs could be separately and unaffected. In fact, the two-hairpin mediated strand displacement can function as a universal joint to connect the trigger and target RNA. In addition to gRNA in the Cas9 system, one can envision that this two-hairpin intermediate joint may also be applied in other nucleic acid systems, such as riboswitch (21–23), RNA interference (15–17), or other Cas systems (54), which will pave a new road for development of RNA-based genetic circuits with combination of advanced nucleic acid computation.

The introduction of the two-hairpin intermediate joint provides a novel approach to establish artificially regulatory connections between naturally occurring endogenous gene expressions. This two-hairpin joint removes the sequence constraint between the trigger RNA X and the target gRNA-Y, so that the target gene Y is independent on RNA X (Figure 1B). This sequence constraint may not be necessarily critical if either the trigger RNA X or the target gene Y is exogeneous so that it can be rationally designed and engineered to share a partially dependent sequence with each other, but does encounter a problem if both of them are endogenous and naturally occurring without sequence correlation. Establishing endogenous genetic connections is of great significance to achieve autonomous control of cellular behaviours with a self-adjusting ability. For instance, if endogenous RNA X exhibits distinct expression profiles in different tissues or developmental stages, linking RNA X with another endogenous target gene Y may impose a regulatory effect on RNA Y expression. One can also envision that if linking a disease-related gene to an independent therapeutic target, selectively manipulating diseased cells without affecting normal cells could also be foreseeable. Using this two-hairpin intermediate joint for conditional control of gRNA in CRISPR/Cas9 function, our current investigations established a primary model that could build a genetic regulation between two sequence-independent endogenous RNAs in both *E. coli* and mammalian cells. Since the trigger RNA is fully unrelated to the target gene, theoretically, by rational design and screening, any endogenous RNA might be selected as a trigger strand to target any desired gene in living cells. Our design would provide a powerful strategy for future investigation and creation of diverse endogenous RNA regulations.

In this work, we have verified that sRNA in *E. coli* and miRNA in human cells can be selected to control endogenous gene expression of mRNA, but notably, future efforts are still needed to see whether all kinds of RNA, such as long non-coding RNA and mRNA with complicated secondary structures, could also be utilized as trigger strands. Besides, all of our designed computing RNAs were genetically encoded into plasmids, and could be also potentially integrated into the cellular genome to permanently build the relationship between previously unrelated RNAs. Moreover, due to the versatility of CRISPR function, the output signal of target gene may not be necessarily limited to CRISPRi/a, but could be other cellular outcomes, such as nucleobase editing (55) and fluorescence imaging (56), with great potential to build a variety of CRISPR-mediated link controlled by endogenous RNA expressions.

Overall, we have employed a two-hairpin mediated strand displacement as a processing joint in a CRISPR/Cas9-based gRNA circuit to manipulate desired gene expressions by independent RNAs in both *E. coli* and mammalian cells. This two-hairpin intermediate joint possesses a sequence-switching machinery, in which a random trigger strand can be processed to release an unconstrained sequence-independent strand and consequently activate the self-inhibitory gRNA for conditional gene regulation. Our strategy can overcome the sequence constraint and achieve autonomous control of endogenous target genes by unrelated endogenous RNAs via programmable CRISPR/Cas9 function. The connection established between endogenous RNAs is a critical step to build the artificial genetic network, in which independent gene expressions could be linked together to generate novel regulatory functions. Introduction of the two-hairpin machine as an RNA processing joint not only greatly broadens the applicable scenarios of CRISPR technology, but also opens a new door for development of other RNA-based genetic circuits to create artificial communications in cells.

## Supplementary Material

gkaa842_Supplemental_File
