# Supplementary material for: Conditional guide RNA through two intermediate hairpins for programmable CRISPR/Cas9 function: building regulatory connections between endogenous RNA expressions
Source: Nucleic Acids Res. 2020 Oct 17;48(20):11773–84. doi: 10.1093/nar/gkaa842 (PMC7672423; doi:10.1093/nar/gkaa842)
Supplement: gkaa842_Supplemental_File [file gkaa842_supplemental_file.pdf]

Supplementary Data for

Conditional guide RNA through two intermediate hairpins for programmable CRISPR/Cas9 function: building regulatory connections between endogenous RNA expressions

Jiao Lin, Yan Liu, Peidong Lai, Huixia Ye, Liang Xu*

MOE Key Laboratory of Bioinorganic and Synthetic Chemistry, School of Chemistry, Sun Yat-Sen University, Guangzhou, 510275, China.

*To whom correspondence should be addressed.
E-mail: xuliang33@mail.sysu.edu.cn

Table of Content

Supplementary Tables 3
Table S1. 3
Table S2. 6
Table S3. 7
Table S4. 8
Table S5. 9
Table S6. 9
Table S7. 10
Table S8. 10
Table S9. 10
Table S10. 11
Table S11. 11
Table S12. 11
Table S13. 12
Supplementary Figures 13
Figure S1. 13
Figure S2. 14
Figure S3. 15
Figure S4. 16
Figure S5. 17
Figure S6. 18
Figure S7. 19
Figure S8. 20
Figure S9. 21
Figure S10. 23
Figure S11. 23
Figure S12. 24

Supplementary Tables

Table S1. Major plasmid constructs in this work.

pJ-dCas9	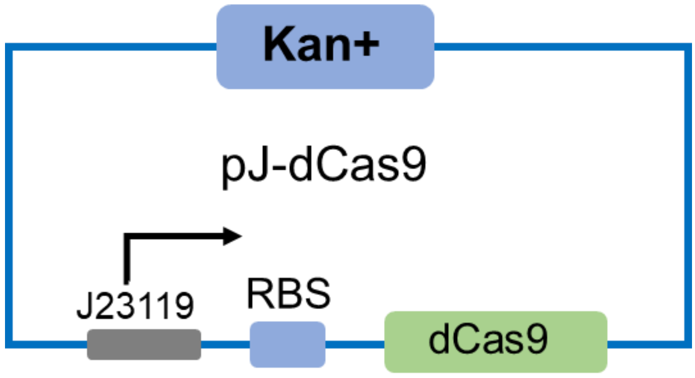
pJ-gRNA-dCas9	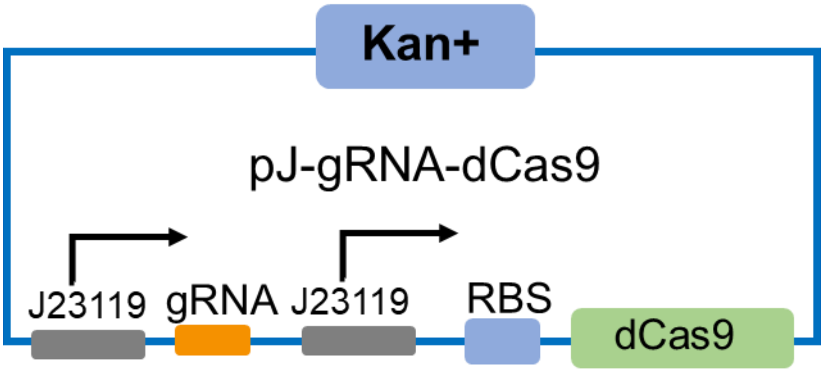
pJ-SH	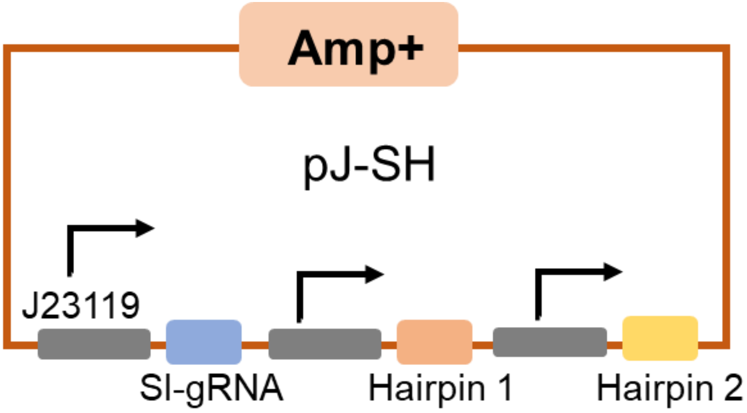

pJ-Tri	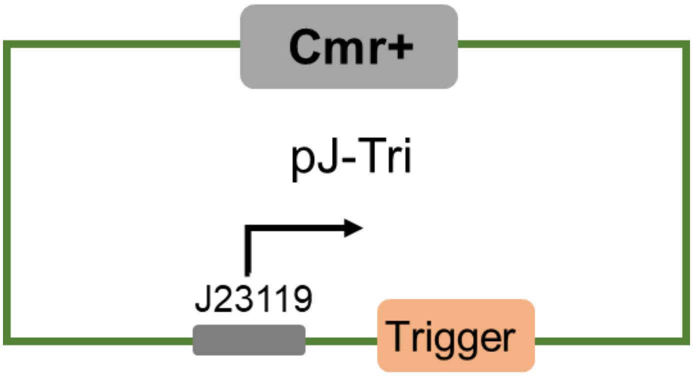
pJ-lac-Tri	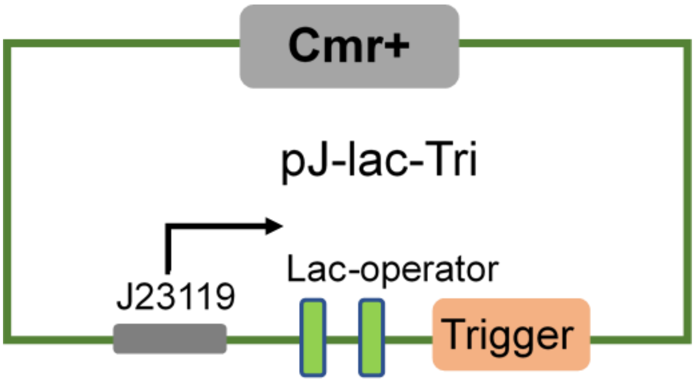
pU6-SH	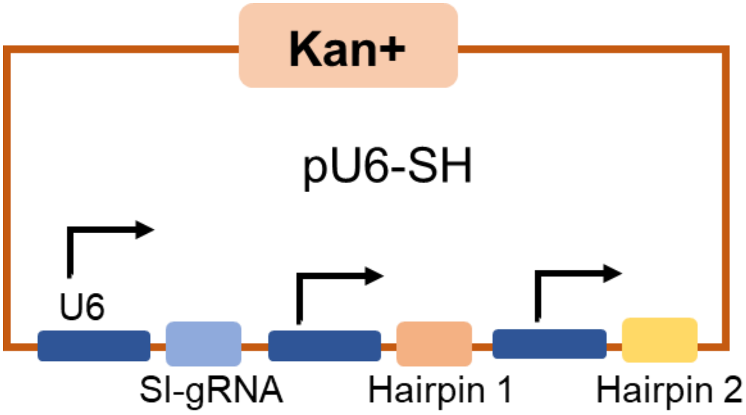
pJ23119	ttgacagctagctcagtcctaggtataatactagt
RBS	tttgtttaactttaagaaggaga
dCas9	atggacaagaagtacagcatcggcctggccatcggcaccaactctgtgggctgggccg<br>tgatcaccgacgagtacaaggtgcccagcaagaaattcaaggtgctgggcaacaccg<br>accggcacagcatcaagaagaacctgatcggcgccctgctgttcgacagcggagaaa<br>cagccgaggccacccggctgaagagaaccgccagaagaagatacaccagacgga<br>agaaccggatctgctatctgcaagagatcttcagcaacgagatggccaaggtggacga<br>cagcttcttccacagactggaagagtccttcctggtggaagaggataagaagcacgagc<br>ggcaccccatcttcggcaacatcgtggacgaggtggcctaccacgagaagtaccccac<br>catctaccacctgagaaagaaactggtggacagcaccgacaaggccgacctgcggct<br>gatctatctggccctggcccacatgatcaagttccggggccacttcctgatcgagggcga<br>cctgaaccccgacaacagcgacgtggacaagctgttcatccagctggtgcagacctac<br>aaccagctgttcgaggaaaaccccatcaacgccagcggcgtggacgccaaggccatc

	ctgtctgccagactgagcaagagcagacggctggaaaatctgatcgcccagctgcccg<br>gcgagaagaagaatggcctgttcggcaacctgattgccctgagcctgggcctgacccccc<br>aacttcaagagcaacttcgacctggccgaggatgccaaactgcagctgagcaaggac<br>acctacgacgacgacctggacaacctgctggcccagatcggcgaccagtacgccgac<br>ctgtttctggccgccaagaacctgtccgacgccatcctgctgagcgacatcctgagagtg<br>aacaccgagatcaccaaggccccccctgagcgcctctatgatcaagagatacgacgag<br>caccaccaggacctgaccctgctgaaagctctcgtgcggcagcagctgcctgagaagt<br>acaaagagattttcttcgaccagagcaagaacggctacgccggctacatcgatggcgg<br>agccagccaggaagagttctacaagttcatcaagcccatcctggaaaagatggacggc<br>accgaggaactgctcgtgaagctgaacagagaggacctgctgcggaagcagcggac<br>cttcgacaacggcagcatcccccaccagatccacctgggagagctgcacgccattctgc<br>ggcggcaggaagatttttacccattcctgaaggacaaccgggaaaagatcgagaagat<br>cctgaccttccgcatcccctactacgtgggccctctggccaggggaaacagcagattcgc<br>ctggatgaccagaaagagcgaggaaaccatcacccccctggaacttcgaggaagtggt<br>ggacaagggcgccagcgcccagagcttcatcgagcggatgaccaacttcgataagaa<br>cctgcccaacgagaaggtgctgcccaagcacagcctgctgtacgagtacttcaccgtgt<br>acaacgagctgaccaaagtgaaatacgtgaccgagggaatgagaaagcccgccttcc<br>tgagcggcgagcagaaaaaagccatcgtggacctgctgttcaagaccaaccggaaag<br>tgaccgtgaagcagctgaaagaggactacttcaagaaaatcgagtgcttcgactccgtg<br>gaaatctccggcgtggaagatcggttcaacgcctccctgggcacataccacgatctgctg<br>aaaattatcaaggacaaggacttcctggacaatgaggaaaacgaggacattctggaag<br>atatcgtgctgaccctgacactgtttgaggacagagagatgatcgaggaacggctgaaa<br>acctatgcccacctgttcgacgacaaagtgatgaagcagctgaagcggcggagataca<br>ccggctggggcaggctgagccggaagctgatcaacggcatccgggacaagcagtcc<br>ggcaagacaatcctggatttcctgaagtccgacggcttcgccaacagaaacttcatgca<br>gctgatccacgacgacagcctgacctttaaagaggacatccagaaagcccaggtgtcc<br>ggccagggcgatagcctgcacgagcacattgccaatctggccggcagccccgccatta<br>agaagggcatcctgcagacagtgaaggtggtggacgagctcgtgaaagtgatgggcc<br>ggcacaagcccgagaacatcgtgatcgaaatggccagagagaaccagaccaccca<br>gaagggacagaagaacagccgcgagagaatgaagcggatcgaagagggcatcaa<br>agagctgggcagccagatcctgaaagaacaccccgtggaaaacacccagctgcaga<br>acgagaagctgtacctgtactacctgcagaatgggcgggatatgtacgtggaccagga<br>actggacatcaaccggctgtccgactacgatgtggacgctatcgtgcctcagagctttctg<br>aaggacgactccatcgataacaaagtgctgactcggagcgacaagaaccggggcaa<br>gagcgacaacgtgccctccgaagaggtcgtgaagaagatgaagaactactggcgcca<br>gctgctgaatgccaagctgattacccagaggaagttcgacaatctgaccaaggccgag<br>agaggcggcctgagcgaactggataaggccggcttcatcaagagacagctggtggaa<br>acccggcagatcacaaagcacgtggcacagatcctggactcccggatgaacactaagt<br>acgacgagaacgacaaactgatccgggaagtgaaagtgatcaccctgaagtccaagc<br>tggtgtccgatttccggaaggatttccagttttacaaagtgcgcgagatcaacaactacca<br>ccacgcccacgacgcctacctgaacgccgtcgtgggaaccgccctgatcaaaaagtac<br>cctaagctggaaagcgagttcgtgtacggcgactacaaggtgtacgacgtgcggaaga<br>tgatcgccaagagcgagcaggaaatcggcaaggctaccgccaagtacttcttctacag<br>caacatcatgaactttttcaagaccgagattaccctggccaacggcgagatccggaagc

dCas9	ggcctctgatcgagacaaacggcgaaacaggcgagatcgtgtgggataagggccgg<br>gactttgccaccgtgcggaaagtgctgtctatgccccaagtgaatatcgtgaaaaagacc<br>gaggtgcagacaggcggcttcagcaaagagtctatcctgcccaagaggaacagcgac<br>aagctgatcgccagaaagaaggactgggaccctaagaagtacggcggcttcgacagc<br>cccaccgtggcctattctgtgctggtggtggccaaagtggaaaagggcaagtccaagaa<br>actgaagagtgtgaaagagctgctggggatcaccatcatggaaagaagcagcttcgag<br>aagaatcccatcgactttctggaagccaagggctacaaagaagtgaaaaaggacctg<br>atcatcaagctgcctaagtactccctgttcgagctggaaaacggccggaagagaatgct<br>ggcctctgccggcgaactgcagaagggaaacgaactggccctgccctccaaatatgtg<br>aacttcctgtacctggccagccactatgagaagctgaagggctcccccgaggataatga<br>gcagaaacagctgtttgtggaacagcacaaacactacctggacgagatcatcgagcag<br>atcagcgagttctccaagagagtgatcctggccgacgctaatctggacaaggtgctgag<br>cgcctacaacaagcacagagacaagcctatcagagagcaggccgagaatatcatcc<br>acctgtttaccctgaccaatctgggagcccctgccgccttcaagtactttgacaccaccatc<br>gaccggaagaggtacaccagcaccaaagaggtgctggacgccaccctgatccacca<br>gagcatcaccggcctgtacgagacacggatcgacctgtctcagctgggaggcgacTA<br>G
Lac-operator	ggaattgtgagcggataacaattcc
U6	gatccgacgcgccatctctaggcccgcgccggccccctcgcacggacttgtgggagaa<br>gctcggctactcccctgccccggttaatttgcatataatatttcctagtaactatagaggctta<br>atgtgcgataaaagacagataatctgttctttttaatactagctacattttacatgataggcttg<br>gatttctataacttcgtatagcatacattatacgaagttataaacagcacaaaaggaaact<br>caccctaactgtaaagtaattgtgtgttttgagactataagtatcccttggagaaccaccttgt<br>tg

Table S2. The DNA sequence (5'-3') used in Figure 2.

RepQ	BHQ-CGAGTGCTCTATGACAAGGGCTAGGTTGAC
RepF	CCCTTGTCATAGAGCACTCG-FAM
Trigger (T)	CGACATCTAACCGGCTAGCTCACTGAC
Hairpin$_1$ (H$_1$)	CGGCTAGCTCACTGACGTCAACCTAGCCCTTGGTCAGTGAGCT<br>AGCCGGTTAGATGTCG
Hairpin$_2$ (H$_2$)	GTCAACCTAGCCCTTGTCATAGAGCACCAAGGGCTAGGTTGAC<br>GTCAGTGAGCTA
Trigger$_{wrong}$ (T$_w$)	GGCGACGTCATTACCCATCCTGGTCGAG

Table S3. The DNA and RNA sequence (5'-3') used in dCas9 binding assay in Figure 3, Figure S3, Figure S4, Figure S5 and Figure S6.

RNA sequence	
gRNA	GGAGUUAGUACCGAAGACCUGUUUUAGAGCUAGAAAUAGCAAGUUAAAAUAAGGCUAGUCCGUUAUCAACUUGAAAAAGUGGCACCGAGUCGGUGC
3'-10-SI-gRNA	GGAGUUAGUACCGAAGACCUGUUUUAGAGCUAGAAAUAGCAAGUUAAAAUAAGGCUAGUCCGUUAUCAACUUGAAAAAGUGGCACCGAGUCGGUGCGAGCAGGUCUUCGGGGGUAAUGACUC
3'-12-SI-gRNA	GGAGUUAGUACCGAAGACCUGUUUUAGAGCUAGAAAUAGCAAGUUAAAAUAAGGCUAGUCCGUUAUCAACUUGAAAAAGUGGCACCGAGUCGGUGCGAGCAGGUCUUCGGUAGGGUAAUGACUC
3'-15-SI-gRNA	GGAGUUAGUACCGAAGACCUGUUUUAGAGCUAGAAAUAGCAAGUUAAAAUAAGGCUAGUCCGUUAUCAACUUGAAAAAGUGGCACCGAGUCGGUGCGAGCAGGUCUUCGGUACUAGGGUAAUGACUC
3'-extended-gRNA	GGAGUUAGUACCGAAGACCUGUUUUAGAGCUAGAAAUAGCAAGUUAAAAUAAGGCUAGUCCGUUAUCAACUUGAAAAAGUGGCACCGAGUCGGUGCGAGCAACUUGAUUAGGGUGAUGGUUCAC
5'-10-SI-gRNA	GGGUAAUGACUCAGGUCUUCGGGGAGUUAGUACCGAAGACCUGUUUUAGAGCUAGAAAUAGCAAGUUAAAAUAAGGCUAGUCCGUUAUCAACUUGAAAAAGUGGCACCGAGUCGGUGC
5'-12-SI-gRNA	GGGUAAUGACUCAGGUCUUCGGUAGGAGUUAGUACCGAAGACCUGUUUUAGAGCUAGAAAUAGCAAGUUAAAAUAAGGCUAGUCCGUUAUCAACUUGAAAAAGUGGCACCGAGUCGGUGC
5'-15-SI-gRNA	GGGUAAUGACUCAGGUCUUCGGUACUAGGAGUUAGUACCGAAGACCUGUUUUAGAGCUAGAAAUAGCAAGUUAAAAUAAGGCUAGUCCGUUAUCAACUUGAAAAAGUGGCACCGAGUCGGUGC
5'-extended-gRNA	GGGAUGCGCCGCUACAUUACGCGUGGAGUUAGUACCGAAGACCUGUUUUAGAGCUAGAAAUAGCAAGUUAAAAUAAGGCUAGUCCGUUAUCAACUUGAAAAAGUGGCACCGAGUCGGUGC
DNA sequence	
dsDNA-1	CGACTACAAGGTGTACGACCAAGGTCTTCGGTACTAACTCCTATAGTGAGTCGTATTAG
FAM-dsDNA-2	FAM-CTAATACGACTCACTATAGGAGTTAGTACCGAAGACCT**TGG**TC

	GTACACCTTGTAGTCG
3'-Trigger	CTGGACGGCGACATCCTGGTCGAG
3'-Hairpin 1	CCTGGTCGAGGTCATTACCCCTCGACCAGGATGTCGCCGTCCAG
3'-Hairpin 2	GTCATTACCCTACCGAAGACCTGGGTAATGACCTCGACCAGG
5'-Hairpin 1	CTCGACCAGGATGTCGCCGTCCAGGAGTCATTACCTGGACGGCG
5'-Hairpin 2	CGCCGTCCAGGTAATGACTCTACCGAAGACCTGAGTCATTAC

Table S4. The gRNA, SI-gRNA$_g$, Trigger, Hairpin 1 and Hairpin 2 sequence (5'-3') used in Figure 4, Figure S8 and Figure S9.

galA (target gene)	
galA-gRNA	**GGCAGGGUAGCCAAAUGCGU**GUUUUAGAGCUAGAAAUAGCAAGUUAAAAUAAGGCUAGUCCGUUAUCAACUUGAAAAAGUGGCACCGAGUCGGUGC
SI-gRNA$_g$	GGCAGGGUAGCCAAAUGCGUGUUUUAGAGCUAGAAAUAGCAAGUUAAAAUAAGGCUAGUCCGUUAUCAACUUGAAAAAGUGGCACCGAGUCGGUGCGAGUACGCAUUUGGCUCCAUCAGUGUAA
Null	GUUUUAGAGCUAGAAAUAGCAAGUUAAAAUAAGGCUAGUCCGUUAUCAACUUGAAAAAGUGGCACCGAGUCGGUGC
Trigger	GGGCGAGGAGGAUAACAUGGCCAU
Hairpin 1(H$_1$) $_g$	UAACAUGGCCAUUUACACUGAUGGAUGGCCAUGUUAUCCUCCUCGCCC
Hairpin 2(H$_2$) $_g$	UUACACUGAUGGAGCCAAAUGCGUCCAUCAGUGUAAAUGGCCAUGUUA
H$_{1w}$	AAGUUGUGCUGGGGGAUUACUGUGCCAGCACAACUUCGCUGUCGCGGU
H$_{2w}$	GGGAUUACUGUGUCGCCCUUGCUCCACAGUAAUCCCCCAGCACAACUU
LacZ (target gene)	
LacZ-gRNA	UUGGGAAGGGCGAUCGGUGCGUUUUAGAGCUAGAAAUAGCAAGUUAAAAUAAGGCUAGUCCGUUAUCAACUUGAAAAAGUGGCACCGAGUCGGUGC
SI-gRNA$_L$	UUGGGAAGGGCGAUCGGUGCGUUUUAGAGCUAGAAAUAGCAAGUUAAAAUAAGGCUAGUCCGUUAUCAACUUGAAAAAGUGGCACCGAGUCGGUGCGAGUGCACCGAUCGCCCCAUCAGUGUAA
Trigger	GGGCGAGGAGGAUAACAUGGCCAU
Hairpin 1(H$_{1L}$)	UAACAUGGCCAUUUACACUGAUGGAUGGCCAUGUUAUCCUCCUCGCCC
Hairpin 2(H$_{2L}$)	UUACACUGAUGGGGCGAUCGGUGCCCAUCAGUGUAA

	AUGGCCAUGUUA
MicF	
MicF-gRNA	AGACAUUCAGAAAUGAAUGAGUUUUAGAGCUAGAAAUAGCAAGUUAAAAUAAGGCUAGUCCGUUAUCAACUUGAAAAAGUGGCACCGAGUCGGUGC

Table S5. The sequence of sRNA mentioned in this paper.

sRNA in E.coil	
MicF	GCUAUCAUCAUUAACUUUAUUUAUUACCGUCAUUCAUUUCUGAAUGUCUGUUUACCCCUAUUUCAACCGGAUGCCUCGCAUCCGGUUUUUUUU
RhyB	GCGAUCAGGAAGACCCUCGCGGAGAACCUGAAAGCACGACAUUGCUCACAUUGCUUCCAGUAUUACUUAGCCAGCCGGGUGCUGGCUUUU

Table S6. The gRNA, SI-gRNA, Hairpin 1 and Hairpin 2 sequence (5'-3') used in Figure 5b, 5c and 5e.

galA (target gene)	
galA-gRNA	Same as galA-gRNA sequence in Table S4
SI-gRNA$_{g}$	Same as galA-gRNA sequence in Table S4
H$_{1Mg}$	ACCGGAUGCCUCUUACACUGAUGGGAGGCAUCCGGUUGAAAUAGGGGU
H$_{2Mg}$	UUACACUGAUGGAGCCAAAUGCGUCCAUCAGUGUAAGAGGCAUCCGGU
H$_{1R}$	ACCCUCGCGGAGUUACACUGAUGGCUCCGCGAGGGUCUUCCUGAUCGC
H$_{2R}$	UUACACUGAUGGGGCGAUCGGUGCCCAUCAGUGUAACUCCGCGAGGGU
lacZ (target gene)	
lacZ-gRNA	Same as lacZ-gRNA sequence in Table S4
SI-gRNA$_{L}$	Same as SI-gRNA$_{L}$ sequence in Table S4
H$_{1ML}$	Same as H$_{1Mg}$
H$_{2ML}$	UUACACUGAUGGGGCGAUCGGUGCCCAUCAGUGUAAGAGGCAUCCGGU

Table S7. Primers (5'-3') used in qPCR assay in E. coil.

**Gene**	F-primer	R-primer	Products size/bp
rssA	AGGACTCATGGCACCTGTTG	ATGCACGCGTGAGGGAAATA	89
LacZ	GCCCATCTACACCAACGTGA	TGAGCGAGTAACAACCCGTC	86
galA	ATTTGGCTACCCTGCCACTC	GCGAACTTTACGGTCATCGC	154
16S RNA	TAATACGGAGGGTGCAAGCG	CTTCGCCACCGGTATTCCTC	200
MicF	GCTATCATCATTAACTTTATTA	AAACCGGATGCGAGGCATC	93

Table S8. The sequence of microRNA mentioned in this paper.

**microRNA in 293T**	
miR17	CAAAGUGCUUACAGUGCAGGUAG
miR16	UAGCAGCACGUAAAUAUUGGCG
Let-7a	UGAGGUAGUAGGUUGUAUAGUU

Table S9. The gRNA, SI-gRNA, Hairpin 1 and Hairpin 2 sequence (5'-3') used in Figure 6.

CXCR4(target gene)	
CXCR4-gRNA	GCAGACGCGAGGAAGGAGGGCGCGUUUAAGAGCUAAGCUGGAAACAGCAUAGCAAGUUUAAAUAAGGCUAGUCCGUUAUCAACUUGAAAAAGUGGCACCGAGUCGGUGCU
SI-gRNA$_{cx}$	GCAGACGCGAGGAAGGAGGGCGCGUUUAAGAGCUAAGCUGGAAACAGCAUAGCAAGUUUAAAUAAGGCUAGUCCGUUAUCAACUUGAAAAAGUGGCACCGAGUCGGUGCUGAGUCGCGCCCUCCUUCGGAUCAGUGUAA
H$_{1-17}$	AGUGCAGGUAGUUACACUGAUCCCUACCUGCACUGUAAGCACUUUG
H$_{2-17}$	UUACACUGAUCCGAAGGAGGGCGCGGAUCAGUGUAACUACCUGCACU
H$_{1-16}$	AAUAUUGGCGUUACACUGAUCCCGCCAAUAUUUACGUGCUGCUA
H$_{2-16}$	UUACACUGAUCCGAAGGAGGGCGCGGAUCAGUGUAACGCCAAUAUU
H$_{1-7a}$	UUGUAUAGUUUUACACUGAUCCAACUAUACAACCUACUACCUCA
H$_{2-7a}$	UUACACUGAUCCGAAGGAGGGCGCGGAUCAGUGUAAAACUAUACAA
H$_{1w}$	UAACAUGGCCAUGAGUCAUUACCGAUGGCCAUGUUAU

	CCUCCUCGCCC
H_{2w}	UAUUAGCGCCGGGUUGUAAAACGACCGGCGCUAAUAU UCACCUAGAUC

Table S10. The gRNA, SI-gRNA, Hairpin 1 and Hairpin 2 sequence (5'-3') used in Figure S12.

ASCL1(target gene)	
ASCL1-gRNA	UGGAGAGUUUGCAAGGAGCGUUUAAGAGCUAAGCUGG AAACAGCAUAGCAAGUUUAAAUAAGGCUAGUCCGUUAU CAACUUGAAAAAGUGGCACCGAGUCGGUGCU
SI-$gRNA_{AS}$	UGGAGAGUUUGCAAGGAGCGUUUAAGAGCUAAGCUGG AAACAGCAUAGCAAGUUUAAAUAAGGCUAGUCCGUUAU CAACUUGAAAAAGUGGCACCGAGUCGGUGCUGAGUCG CUCCUUGCAAAGGAUCAGUGUAA
H_{1AS}	AGUGCAGGUAGUUACACUGAUCCCUACCUGCACUGUA AGCACUUUG
H_{2AS}	UUACACUGAUCCUUUGCAAGGAGCGGAUCAGUGUAAC UACCUGCACU
H_{1w}	Same as H_{1w} sequence in Table S9
H_{2w}	Same as H_{2w} sequence in Table S9

Table S11. Primers (5'-3') used in qPCR assay in HEK293T cells.

Gene	F-primer	R-primer	Product size/bp
GAPDH	ACAGTCAGCCGCATCTTCTT	ACGACCAAATCCGTTGACTC	186
ASCL1	GGAGCTTCTCGACTTCACCA	AACGCCACTGACAAGAAAGC	125
CXCR4	CCCTTGGAGTGTGACAGCTT	TTGTGGGTGGTTGTGTTCCA	113

Table S12. Stem-loop Primers (5'-3') used in microRNA-cDNA synthesis.

Micro RNA	Stem-Loop primer
miR17	GTCGTATCCAGTGCAGGGTCCGAGGTATTCGCACTGGATACG ACCTACCT
miR16	GTCGTATCCAGTGCAGGGTCCGAGGTATTCGCACTGGATACG ACCGCCAA
Let-7a	GTCGTATCCAGTGCAGGGTCCGAGGTATTCGCACTGGATACG ACAACTAT

Table S13. Primers (5'-3') used in microRNA qPCR assay

Gene	F-primer	R-primer
U6	CTCGCTTCGGCAGCACA	AACGCTTCACGAATTTGCGT
miR17	GCGCAAAGTGCTTACAGTGC	AGTGCAGGGTCCGAGGTATT
miR16	CGCGTAGCAGCACGTAAATA	AGTGCAGGGTCCGAGGTATT
Let-7a	GCGCGTGAGGTAGTAGGTTGT	AGTGCAGGGTCCGAGGTATT

Supplementary Figures

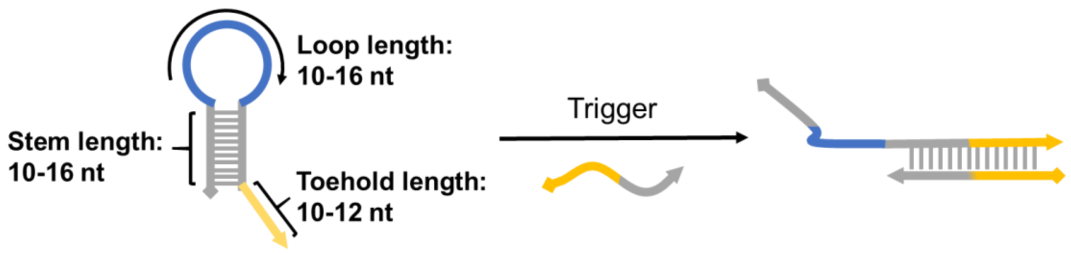

Figure S1. Design of the hairpin structure. For the hairpin structures, the length of the stem and loop varies from 10-nt to 16-nt depending on the trigger strand and the GC content of the sequence according to the calculation by NUPACK. The length of toehold is an important point for efficient stand displacement. The length of toehold could not be either too short or too long. A short toehold would not be efficiently initiate the strand displacement, whereas the long the toehold would bind to nonspecific strands to inhibit the designed displacement. Herein, the toehold design was restricted between 10-12 nt, as previous investigations suggested this length is necessary for efficient strand displacement in living cells (see ref 21 in the main text); in the meantime, the interaction between the toehold and nonspecific strands could be minimized. Taking the typical RNA hairpin with a 12-nt toehold and a 12-nt stem and loop as an example, the ΔG value at 37 °C calculated by NUPACK was about -19 kcal/mol for the sequence with 50% GC content; after the strand displacement, the ΔG value for the duplex structure was about -44 kcal/mol (hybridization between 1 μM hairpin and 1 μM trigger). The ΔΔG value between this structural switch was about -25 kcal/mol, indicating a thermodynamically favored strand displacement process.

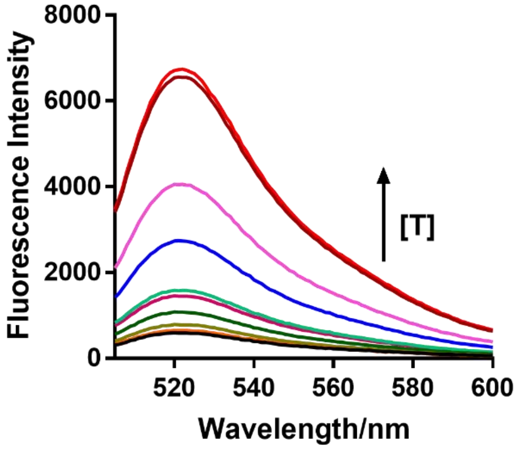

Figure S2. Fluorescence signal was gradually enhanced along with the increasing concentration of trigger strand T. [T] =0, 6, 12, 18, 24, 30, 45, 60, 75, and 90 nM, respectively. The computing machinery and the reporter system was described in Figure 2a.

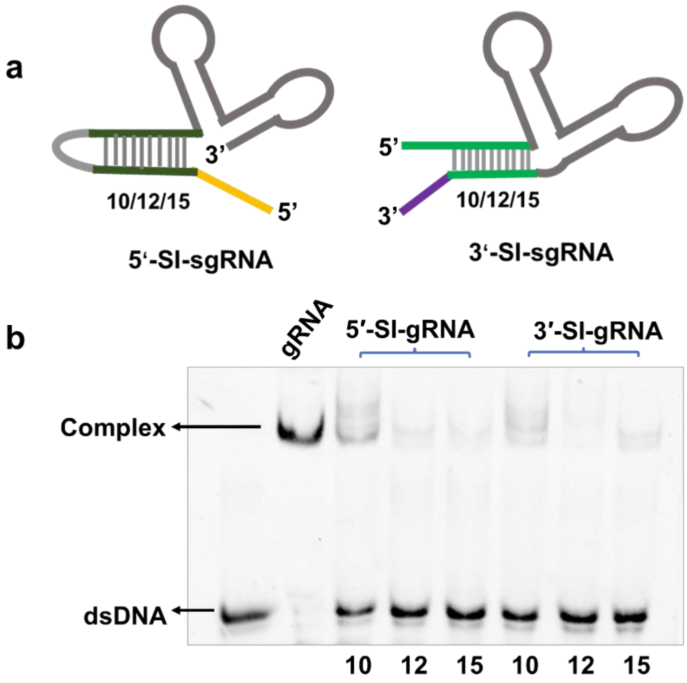

Figure S3. Both 5’-SI-gRNA and 3’-SI-gRNA inhibited dCas9 binding activity. (a) Sketch for the design of 5’-SI-gRNA and 3’-SI-gRNA. The number 10, 12 and 15 represented the length of base pairing between the guide region and the extended strand from 5’- or 3’-end of gRNA. (b) Inhibited complex formation with 5’-SI-gRNA and 3’-SI-gRNA. Clearly, a 12-bp RNA duplex would be stable enough to prevent the dCas9 protein from binding with dsDNA.

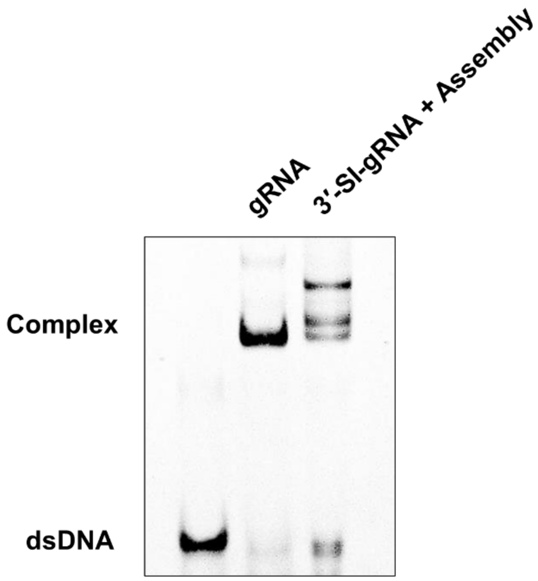

Figure S4. Binding behavior of dCas9/gRNA annealed with the duplex assembly at the 3’-end. In this experiment, the 3’-SI-gRNA was annealed with the excessive trigger strand and two hairpins to form a defined duplex assembly at the 3’-end. As clearly shown in this gel shift assay, dCas9 and the gRNA with the duplex assembly formed multiple complex bands when bound to the DNA substrate. There multiple bands were potentially caused by non-specific interactions between dCas9 and the duplex assembly paired at the 3′-end of gRNA.

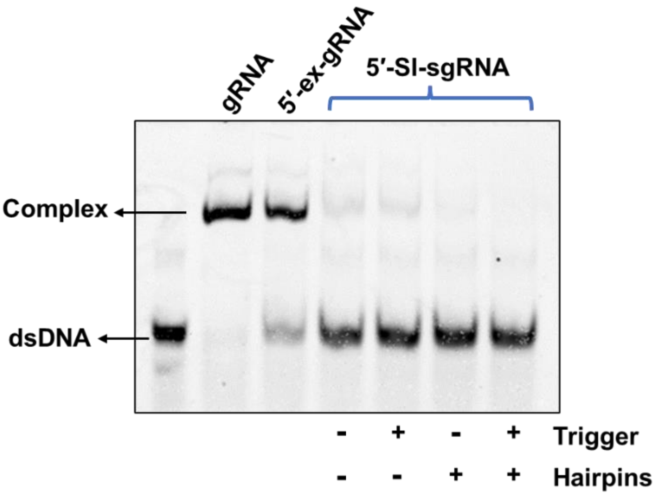

Figure S5. 5’-SI-gRNA could not efficiently recover by the computing assembly. The 5’-ex-gRNA represents the 5’ extended gRNA without complementary sequence against the guide region. Sequences used in this binding assay were listed in Supplementary Table S3.

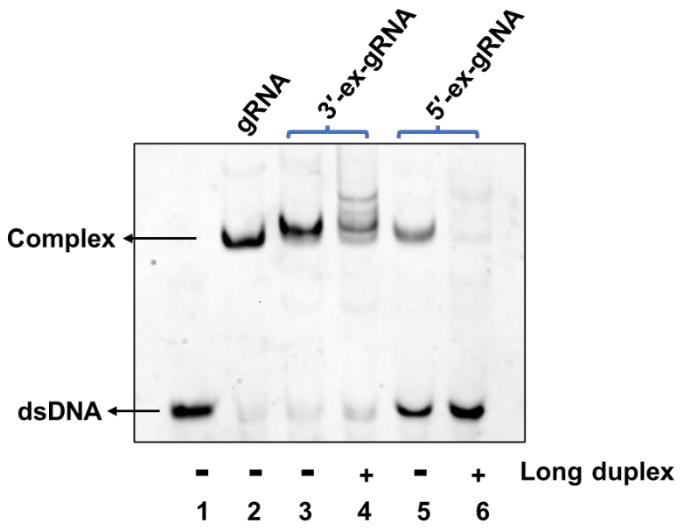

Figure S6. The binding ability of dCas9 is hardly influenced by the 3'-extension of gRNA but significantly diminished by the prolonged 5'-extension of gRNA. lane 1: FAM-labeled dsDNA substrate; lane 2: the unmodified gRNA bound with dCas9; lane 3: the binding behavior of dCas9 with the 3'-ex-gRNA (elongated with a 24 nt single strand sequence at 3'-end that does not pair with the conserved region of gRNA); lane 4: the binding behavior of dCas9 with the 3'-ex-gRNA paired with a long duplex (59 bp) at 3'-extended region to mimic the binding of the nucleic acid assembly; lane 5: the binding behavior of dCas9 with 5'-ex-gRNA (elongated with a 24 nt single strand sequence at 5'-end that does not pair with the conserved region of gRNA); lane 6: the binding behavior of dCas9 with the 5'-ex-gRNA paired with a long duplex (59 bp) at 5'-extended region to mimic the binding of the nucleic acid assembly.

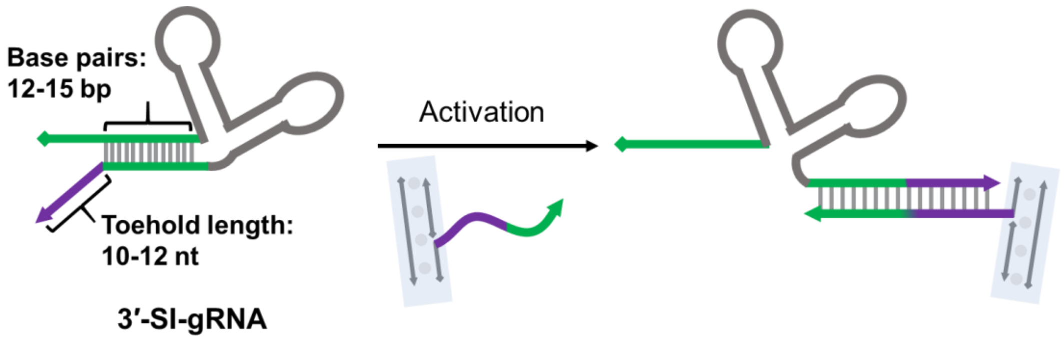

Figure S7. Design of the 3'-SI-gRNA structure. According to the binding analysis of dCas9 (Figure S3), the 3'-extended domain of gRNA must form a 12-15 bp duplex from the seed region of guide sequence to fully inhibit the dCas9 activity. After the formation of inhibited gRNA, a 10-12 nt toehold is left at the 3'-end for initiation of strand displacement. In a way similar to the toehold design for the hairpin structure, the toehold left for 3'-SI-gRNA could not be either too short or too long. A 10-12 nt toehold was selected accordingly. Taking the typical 3'-SI-gRNA with a 12-nt toehold and a 12-bp duplex as an example, the ΔG value at 37 °C calculated by NUPACK was about -39 kcal/mol for the extended sequence with 50% GC content; after the strand displacement, the ΔG value of the active gRNA with the duplex structure was about -69 kcal/mol (hybridization between 1 μM SI-gRNA and 1 μM activating strand). The ΔΔG value between this structural switch was about -30 kcal/mol, indicating a thermodynamically favored strand displacement process.

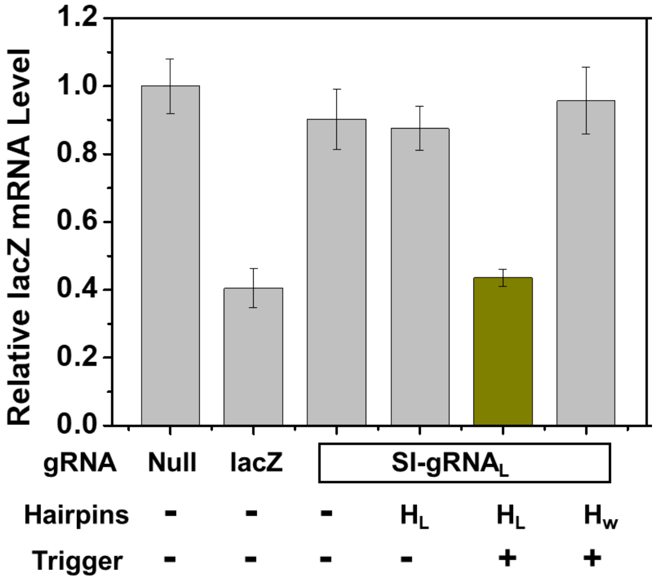

Figure S8. An independent exogenous RNA to control the expression of endogenous gene lacZ in E. Coli. gRNA that could not target any genes as negative control (Null); The unmodified gRNA that could directly target lacZ gene as positive control (lacZ). H_L represents the two computing hairpins that used to link the trigger RNA with LacZ gene, whereas H_w represents incorrect hairpins that could not activate the computing process. Error bars were standard deviations derived from at least three biological replicates.

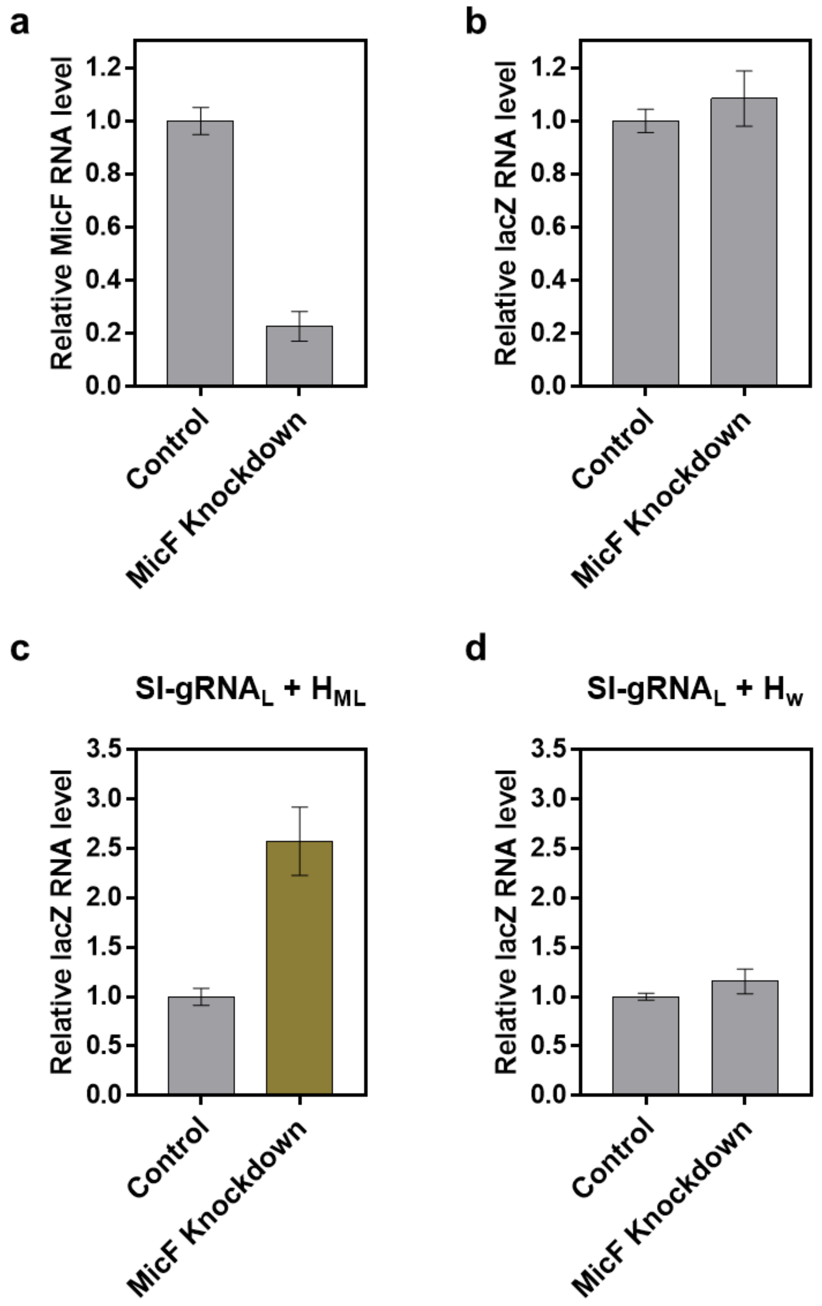

Figure S9. Knockdown of MicF gene can influence the expression level of lacZ RNA through the two-hairpin intermediate joint coupled with CRISPR/Cas9 function. (**a**) Knockdown effect of MicF gene by CRISPRi in *E. coli*. (**b**) Knockdown of MicF gene could hardly affect the lacZ RNA expression in the absence of any connecting elements. (**c**) Knockdown of MicF gene significantly upregulated the expression of lacZ gene in the presence of two hairpins (H_{ML}) and SI-gRNA$_L$. (**d**) Knockdown of MicF gene could hardly affect the expression of lacZ gene with incorrect hairpins (H_w). Two plasmids were

transfected into E. coli: pJ-gRNA-dCas9 and pJ-SH. The gRNA expressed by pJ-gRNA-dCas was the standard gRNA that can target the MicF gene (see Table S4 for the sequence information) to knockdown its transcriptional level. The control sample indicated that the gRNA expressed by pJ-gRNA-dCas9 could not target any site in the MicF gene. The pJ-SH was utilized to express hairpins and SI-gRNA. The SI-gRNA$_L$ was designed to regulate the lacZ gene. In the control samples, the expression of MicF gene can activate the SI-gRNA$_L$ through the two-hairpin intermediate joint (H$_{ML}$) to target the lacZ gene, and therefore, the lacZ gene was repressed by CRISPR function. When the MicF gene was knocked down, the activation of SI-gRNA$_L$ was significantly reduced, and consequently, the lacZ gene was upregulated compared to the repressed state as shown in the panel **c**. However, if the hairpins were incorrect (H$_w$ in the panel **d**), the expression of lacZ gene could not be associated by the variation of MicF gene. The plasmid information was listed in Table S1. Error bars were standard deviations derived from at least three biological replicates.

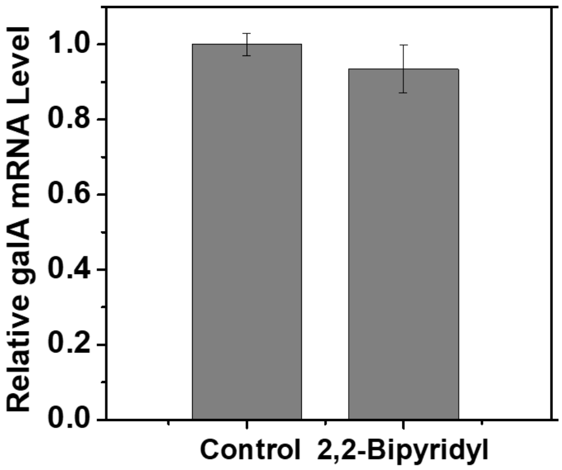

Figure S10. The expression level of galA mRNA was hardly affected by treatment of 2,2-bipyridyl in E. Coli without computing hairpins. E. Coli was treated with 0.6 mM 2,2-bipyridyl and compared with the untreated sample. Error bars were standard deviations derived from at least three biological replicates.

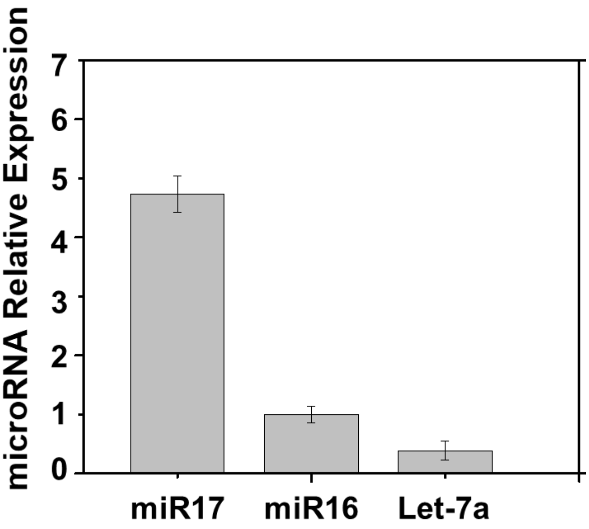

Figure S11. Relative miRNA expression levels of miR17, let-7a and miR16. Error bars were standard deviations derived from at least three biological replicates.

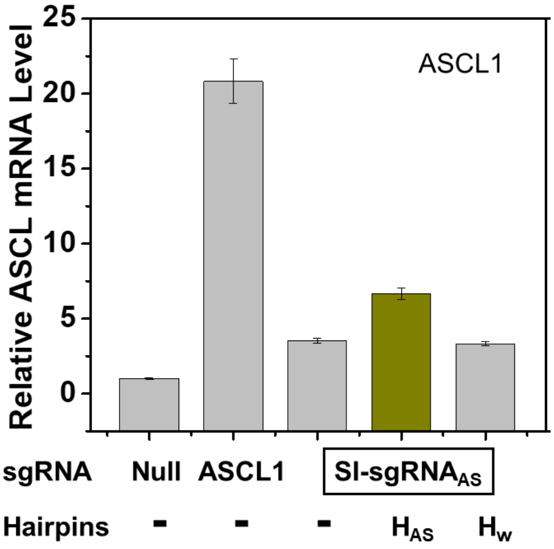

Figure S12. The ASCL1 gene can be linked with miR17 by intermediate joint hairpins. gRNA that could not target any genes as negative control (Null); the unmodified gRNA that could directly target ASCL1 gene to up-regulate the expression level as positive control (ASCL1). The self-inhibited gRNA targeting ASCL1 (SI-gRNA$_{AS}$) was only activated by miR17 in the presence of correct computing hairpins (H$_{17\text{-}AS}$) but not the wrong hairpins (H$_{w}$). Notably, the activating function on the ASCL1 gene was relatively less effective than that on CXCR4, which was probably attributed to different sensitivities towards the released CRISPR function in the different positions and environments of chromosome. Error bars were standard deviations derived from at least three biological replicates.
